# Paediatric dominant and non-dominant handgrip reference curves and the association with body composition

**DOI:** 10.1080/03014460.2023.2298474

**Published:** 2024-01-22

**Authors:** Catherine M. Avitabile, David R. Weber, Babette S. Zemel

**Affiliations:** a Deptartment of Paediatrics, University of PA Perelman School of Medicine, Philadelphia, PA, USA; b Division of Cardiology, Children’s Hospital of Philadelphia, Philadelphia, PA, USA; c Division of Endocrinology, Children’s Hospital of Philadelphia, Philadelphia, PA, USA; d Division of Gastroenterology, Hepatology, and Nutrition, Children’s Hospital of Philadelphia, Philadelphia, PA, USA

**Keywords:** Muscle, densitometry, children, NHANES

## Abstract

**Background:**

Lack of paediatric reference data limits the utility of handgrip strength as a measure of fitness and well-being.

**Aim:**

To develop paediatric handgrip reference curves and evaluate associations with body size and composition and race/ethnicity group.

**Subjects and methods:**

Handgrip, body size and composition data were obtained from National Health and Nutrition Examination Survey 2011–2014 participants aged 6–20 years. Densitometry-derived fat and appendicular lean soft tissue mass index Z-scores (FMIZ, ALSTMIZ) were generated in participants >8 years. Dominant and non-dominant handgrip reference curves were created using the LMS method. Analyses included sample weights to produce nationally representative estimates.

**Results:**

Differences in handgrip strength according to hand dominance increased with age. Handgrip strength was associated with height and arm length Z-scores (*R* = 0.42 to 0.47) and ALSTMIZ (*R* = 0.54). Handgrip strength was higher in the non-Hispanic Black group and lower in the Mexican American compared to non-Hispanic White group. Group differences were attenuated when adjusted for height, arm length or ALSTMIZ.

**Conclusion:**

Paediatric handgrip reference curves were generated from which individual Z-scores can be calculated separately for dominant versus non-dominant hand and adjusted for body size. Association with ALSTMIZ suggests handgrip Z-score may be used as a measure of functional body composition.

## Introduction

Muscular fitness is associated with improved bone health, decreased metabolic and cardiovascular disease, and overall wellness in children (Smith et al. 2014). Improvements in muscular strength also complement improvements in aerobic fitness, contributing to overall health benefits of regular physical activity (Atlantis et al. 2009; Faigenbaum et al. 2013; Cadore et al. 2014). Grip strength, measured by handheld dynamometer, is an accessible, inexpensive, and accurate measurement of muscle fitness and strength. In population studies, it provides information about overall fitness and health (Yuki et al. 2023; de Souza et al. 2022), and in the clinical setting, it is useful for assessing effects of disease processes, medications, or behaviour such as physical inactivity on muscle mass and function (Dougherty et al. 2011; Hogan et al. 2020; Meyer et al. 2022; de Koning et al. 2023). However, the utility of handgrip strength measures in children is limited by a lack of adequate reference data from which to calculate Z-scores relative to age and gender with adjustment for body size.

Some previous studies have described normative values of handgrip strength by age and gender for populations of healthy children across the world (Catley and Tomkinson 2013; Omar et al. 2015; Little 2017; Lim et al. 2019), but these studies were based on small sample sizes, limited age ranges, or did not assess the relationship between handgrip strength and body size. Handgrip strength data collected as part of the U.S. National Health and Nutrition Examination Survey (NHANES) offers an opportunity to overcome these short-comings. Prior publications have reported average estimates of paediatric handgrip strength by age, gender, and body size using NHANES data (Ervin et al. 2013, 2014; Laurson et al. 2017; Kocher et al. 2019; McGrath et al. 2020); however, these studies have significant limitations. First, some studies reported only a subset of the paediatric population within a limited age range or from a single NHANES survey (Ervin et al. 2013, 2014; Laurson et al. 2017). Second, some prior studies (Laurson et al. 2017; Kocher et al. 2019) reported reference values but did not apply sample weights as recommended by the National Centre for Health Statistics (NCHS) when using NHANES data so their results do not accurately represent the U.S. population. Third, no existing paediatric publication included smoothed percentile curves from which individual Z-scores can be calculated. Kocher et al. (2019) reported mean and standard deviation grip strength by age group and percentile, but the percentiles were not smoothed. Analytic techniques that provide smoothed reference percentiles avoid random fluctuations in percentile distributions, especially at the extremes of the distribution (Flegal 1999). While McGrath et al. (2020) published smoothed percentile curves of handgrip, the investigators did not provide the data from which to calculate individual level Z-scores. Finally, several prior studies reported maximum combined (left and right hand) handgrip strength (Ervin et al. 2013, 2014; Laurson et al. 2017) and did not differentiate dominant from non-dominant hand. The effect of hand dominance on grip strength in youth has been demonstrated previously (Lim et al. 2019). Wang et al. (2019) observed that grip strength was about 5% greater on the dominant vs. non-dominant hand and the differences were significantly greater for right-hand dominant individuals versus left-hand dominant individuals in a large sample of participants ages 6–80 years across two NHANES surveys. McGrath et al. (2020) reported percentile curves according to hand dominance, noting that hand dominance can change across the lifespan and that grip strength may not always be greater in the dominant hand (Sivagnanasunderam et al. 2015; Wang et al. 2018). However, the paper does not include the data from which to calculate individual level Z-scores by hand dominance. In children, neuromuscular, orthopaedic, or developmental disorders may produce asymmetries and affect handedness, supporting the differentiation of grip strength Z-scores by hand dominance for the most accurate description of overall muscle strength in individual youth.

Additionally, previous studies have examined associations of weight or body mass index (BMI) with handgrip strength (Ervin et al. 2014; Laurson et al. 2017; Kocher et al. 2019; McGrath et al. 2020). However, weight and BMI may conceal differences in fat and lean mass distribution in certain populations (Harsha et al. 1978; Schutte et al. 1984; Goran et al. 1997; Nelson and Barondess 1997; Lu et al. 2011; Sousa et al. 2013; Weber et al. 2013; Marwaha et al. 2014). For example, appendicular lean soft tissue index, a measure of skeletal muscle in the arms and legs, is significantly less in youth who identify as Asian and significantly higher in youth who identify as African American, compared to those who identify as White (Zemel et al. 2023). Muscle strength scales to muscle size and to the length of bone over which muscle forces are exerted. As such, handgrip strength may be a useful indicator of lean body mass or muscle function, and group differences in muscle strength may be related to body composition differences. No prior studies have examined associations of body composition measures with handgrip strength – an important consideration to support handgrip strength as a potential indicator of functional body composition (Lad et al. 2013).

Similarly, height as an indicator of bone length is an important determinant of handgrip strength but the association differs by age and sex (Kocher et al. 2019). In children, height and age co-vary, so handgrip strength-for-age encapsulates height effects to a large degree. Relative to handgrip strength-for-age, handgrip strength relative to height-for-age may be a more meaningful indicator since it captures residual size effects of height as well as pubertal maturation, since earlier and later maturing children will differ in their height-for-age during the peripubertal years. The effects of pubertal maturation on body composition are well-recognised (Zemel 2022). Assessment of muscle strength relative to height-for-age may be especially valuable in the clinical evaluation of individuals with altered body size, maturation or body composition.

In light of these gaps in the literature we aimed to: 1) generate smoothed age- and gender-specific paediatric handgrip reference distributions for dominant and non-dominant hand using appropriate sampling weights and the LMS (Lambda, Sigma, Mu) method (Cole and Green, 1992) to facilitate calculation of Z-scores (D-HGZ, ND-HGZ); 2) develop size adjustment factors for handgrip strength Z-scores using height-for-age Z-score (HAZ) and upper arm length Z-score (UALZ); 3) evaluate associations between handgrip strength Z-scores and densitometry (DXA)-derived lean body mass index Z-score (LBMIZ) and appendicular lean soft tissue mass index Z-scores (ALSTMIZ), and explore differences in handgrip strength among subgroups of the NHANES sample.

## Methods

### Study sample

**Figure 1.**
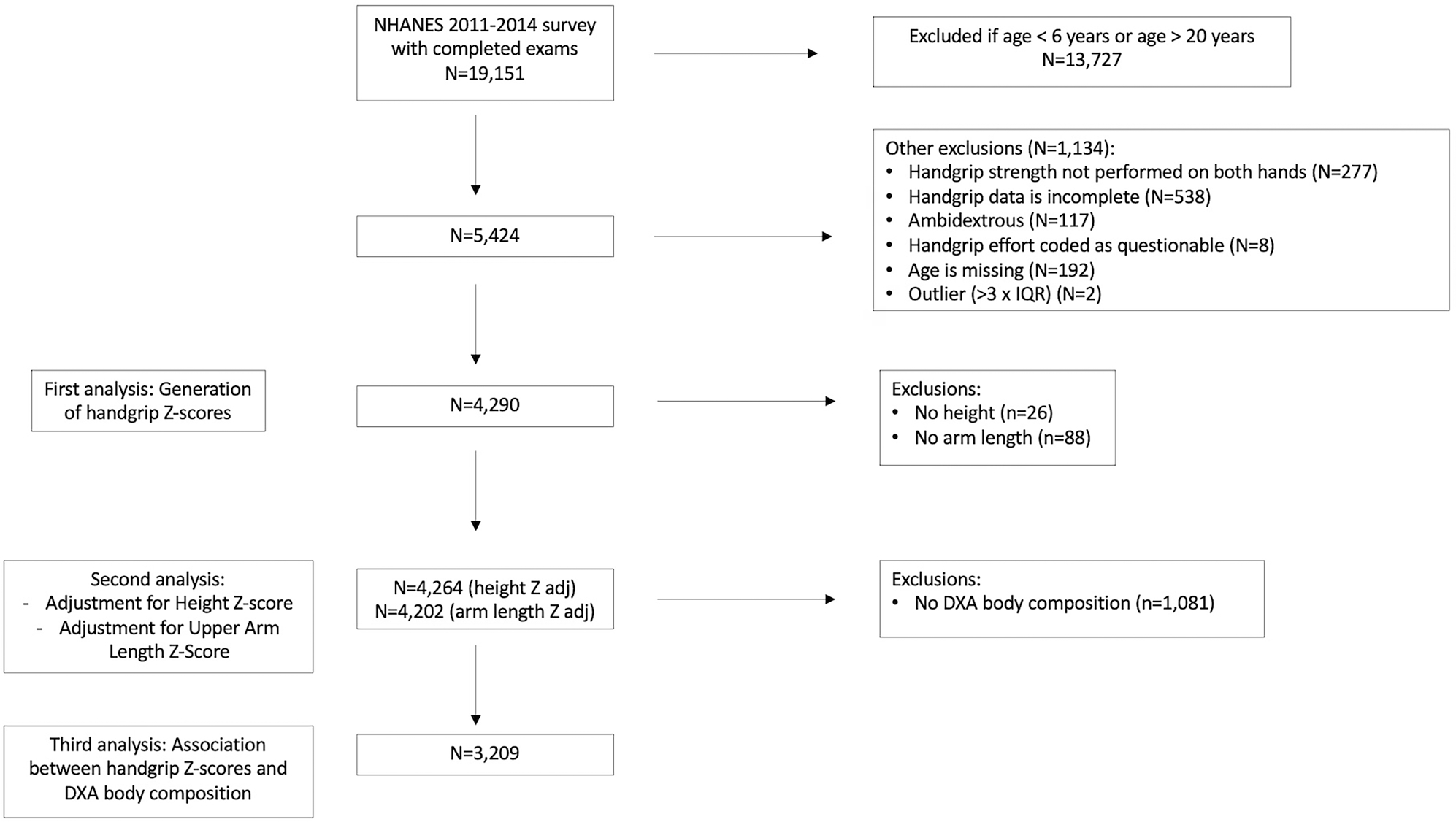
Participant flow chart.

This study utilised publicly available cross-sectional handgrip and body composition data from NHANES 2011–2014 participants aged 6–19.9 years (Figure 1). All available data were used. NHANES is an annual survey conducted by the NCHS that uses a complex, multi-stage probability sampling method designed to produce a dataset representative of the noninstitutionalized U.S. civilian population (National Center for Health Statistics 2014). The survey included a household interview and a detailed examination obtained in mobile examination centres. Approval for NHANES 2011–2014 was obtained from the NCHS Research Ethics Review Board.

For the generation of handgrip and upper arm length Z-scores, we included participants ages 6–19.9 years with bilateral handgrip measurements. Participants were excluded for the following reasons: lack of bilateral measurements, incomplete handgrip data or questionable handgrip effort, ambidextrous status, missing age, or outlier status for handgrip strength (no outliers were identified for upper arm length). Outliers were identified by calculating the interquartile range by sex and integer age years and sex, and flagging values that were more than three times the interquartile range below the 25th percentile or above the 75th percentile. Sub-analyses were performed on participants with DXA data.

### Study data

Dominant and non-dominant handgrip strength was determined using a Takei Digital Grip Dynamometer, Model T.K.K.5401. Participants self-reported handedness and were instructed to perform warm-up exercises prior to the procedure. Each hand was tested three times, alternating hands between trials with a 60-second rest between measurements on the same hand. The maximum handgrip strength for each hand was used for analysis. Full details of the handgrip strength methodology are available in the NHANES Muscle Testing Procedures Manual (National Center for Health Statistics 2011a).

Age was calculated in months as reported at the time of examination. U.S. Census Bureau classifications for race and Hispanic origin were ascertained by participant self-report at the time of the interview. These were categorised in the NHANES database as: Mexican American, Other Hispanic, Non-Hispanic White, Non-Hispanic Black, Other Race (including multi-racial) and missing. Height (cm) and weight (kg) were obtained by using standard procedures and were used to calculate BMI (kg/m^2^). Sex-specific height, weight, and BMI Z-scores for age were calculated by using the 2000 CDC reference data (Kuczmarski et al. 2000). Upper arm length was measured with a tape measure (0.1 cm) and the arm bent to 90° at the elbow. The measurement was taken from the uppermost edge of the right acromion process to the tip of the olecranon along the centre of the posterior surface of the upper arm (National Center for Health Statistics 2011b). Fat mass index, lean body mass (excluding bone), and appendicular lean soft tissue mass (ALSTM, excluding bone) were obtained from whole body DXA scans. DXA scans were performed in participants over 8 years of age using a Hologic A densitometer and analysed using APEX software version 4. Fat mass index (FMI), lean body mass index (LBMI), and appendicular lean soft tissure mass index (ALSTMI) were calculated [(kg)/height (m)^2^] and converted into age- and sex-specific Z-scores (Zemel et al. 2023). The NHANES body composition adjustment applied by the NCHS (Schoeller et al. 2005) was removed by dividing lean body mass by 0.946 and adjusting fat mass accordingly to maintain whole body mass in order to match the Zemel reference dataset.

### Generation of handgrip and upper arm length reference curves

Sex-specific reference curves for dominant and non-dominant handgrip and upper arm length were generated using the LMS method described by Cole and Green (1992) and implemented in R programming language using the Generalised Additive Models for Location, Scale, and Shape (GAMLSS library) in R (Stasinopoulos and Rigby 2007). The LMS method generates smoothed growth curves using the Box-Cox Cole and Green (BCCG) transformation. Model selection was performed sequentially by gradually increasing the degrees of freedom starting with *mu* (M), and then moving on to the *lambda* (L) and *sigma* (S) model parameters. Model selection was based on several considerations including the Akaike Information Criterion (AIC), diagnostic plots (e.g. residual, worm and trend plots) and visual inspection of the curves to avoid overfitting. Age-specific values for the power (L), median (M), and coefficient of variation (S) were estimated and used to calculate exact centile curves using Equation 1:

(1) $$\text{Centile}\;=M(1+LSZ{)}^{1/L}$$

where L,M, and S are age-specific values, and Z is the value of a given percentile in the cumulative standard normal distribution (e.g. Z=0 for the 50^th^ centile). Exact Z-scores (Z) can be calculated for a measurement (X), using the age-specific L,M, and S parameters and Equation 2:

(2) $$Z=\left[(X/M{)}^{L}-1\right]/LS$$

### Statistical analysis

Statistical analyses were conducted with STATA 16.1 (StataCorp LLC). Survey data commands using the “svy” prefix in STATA were used to account for the multi-stage NHANES sample design and included sample weights as recommended by the NCHS to produce estimates representative of the U.S. population (Johnson et al. 2014). Standard descriptive statistics were used to summarise demographic characteristics and outcome data. Sex-specific regression analyses examined associations between indicators of body size for age (HAZ and UALZ) and D-HGZ and ND-HGZ. Interaction terms were included to consider whether the effect of body size varied as a function of age (e.g. HAZ × Age interaction). Based on these analyses, we developed adjustment equations for handgrip strength that adjusted for HAZ or UALZ as previously described (Zemel et al. 2010, 2011). This two-step method first involves predicting D-HGZ or ND-HGZ with age- and sex-specific prediction equations using either HAZ or UALZ as follows:

(3) PredictedHandgripZ=a+b*HAZ(orUALZ),

where a and b are age- and sex-specific coefficients.

The size adjusted Handgrip Z is the difference between the age and sex specific Handgrip Z and the Predicted Handgrip Z calculated as follows:

(4) HAZ(orUALZ)adjustedHandgripZ=HandgripZ-PredictedHandgripZ.

Pearson correlation coefficients were determined using the “corr_svy” STATA command, a user-written command compatible with “svy” that is available for download via the “findit” command, to describe associations between handgrip strength and continuous pre-specified outcomes of interest while accounting for NHANES sample design. Multiple linear regression models were developed to investigate the effect of gender, self-identified race/ethnicity group, and body composition on handgrip strength. Interaction terms were included to assess for differences in the strength of associations between gender and racial/ethnic groups. Variance inflation factors of all response and predictor variables were checked to investigate for collinearity. Akaike information criterion were considered to inform selection of final models.

## Results

### Dominant and non-dominant handgrip reference curves

Descriptive characteristics of the NHANES sample population used to create handgrip reference curves are shown in Table 1, including the United States population weighted proportions of gender and self-identified race/ethnicity groups. Handgrip was assessed on 4290 participants, and DXA measurement of lean mass and fat mass was available on 76% (*n* = 3244) and 75% (*n* = 3219) of the NHANES sample, respectively. Characteristics of the 3209 participants with complete handgrip, body size, and DXA data are available elsewhere (Supplemental Table 1).

**Figure 2.**
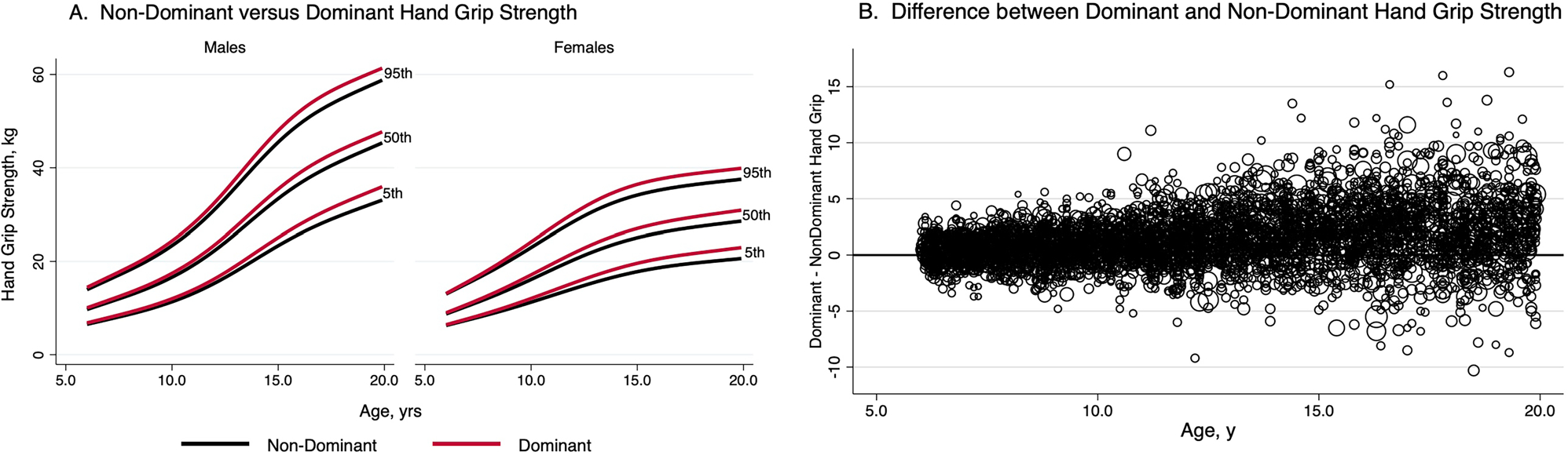
Reference curves for dominant and non-dominant handgrip by gender (A) and difference between dominant and non-dominant handgrip with age (B).

Smoothed reference percentiles for dominant and non-dominant handgrip and upper arm length in males and females aged 6–19.9 years are shown in Tables 2 and 3. In addition to the 50th percentile “M,” each table also provides the L and S values, which can be used to calculate Z-scores for individuals. Reference percentiles for handgrip according to age in tenths of a year are available elsewhere (Supplemental Tables 2 and 3). Reference curves illustrating the 5th, 50th, and 95th centiles for dominant and non-dominant handgrip in males and females are shown in Figure 2(a). Males have noticeably greater handgrip strength then females from early adolescence onward, and the age-related increase plateaus in older females, whereas it continues to increase in older males. The difference between dominant and non-dominant handgrip increases with age and becomes increasingly variable as shown in Figure 2(b).

### Associations of anthropometry and body composition on handgrip Z-scores

Handgrip Z-scores were significantly associated with Z-scores for height, BMI, upper arm length, lean body mass index, appendicular lean soft tissue mass index, and fat mass index (Table 4). Among body composition variables, the correlations between handgrip Z-scores and ALSTMIZ were the highest (*r* = 0.54, *p* < .001 for ND-HGZ and D-HGZ). The correlations between handgrip Z-scores and FMIZ were the lowest (*r* = 0.17, *p* < .001 for both ND-HGZ and D-HGZ).

Using sex-stratified regression analyses with interaction terms for HAZ (or UALZ) × age (in whole year intervals), we developed equations to predict D-HGZ and ND-HGZ based on body size as described in Equation 3 above. The prediction equations are shown in Table 5. The predicted Z-score from Equation 3 is used in Equation 4 to calculate a size adjusted *Z* score for handgrip strength which represents the difference between a child’s handgrip Z for age and the predicted *Z* based on their size. For example, for a 10 year old male with HAZ = 2.98 and D-HGZ = 1.58, his predicted D-HGZ is 1.60 [−0.248 + (.619 × 2.98)], and his HAZ-adjusted D-HGZ is −0.02 [1.58 – 1.60].

### Group differences in handgrip Z-scores

**Figure 3.**
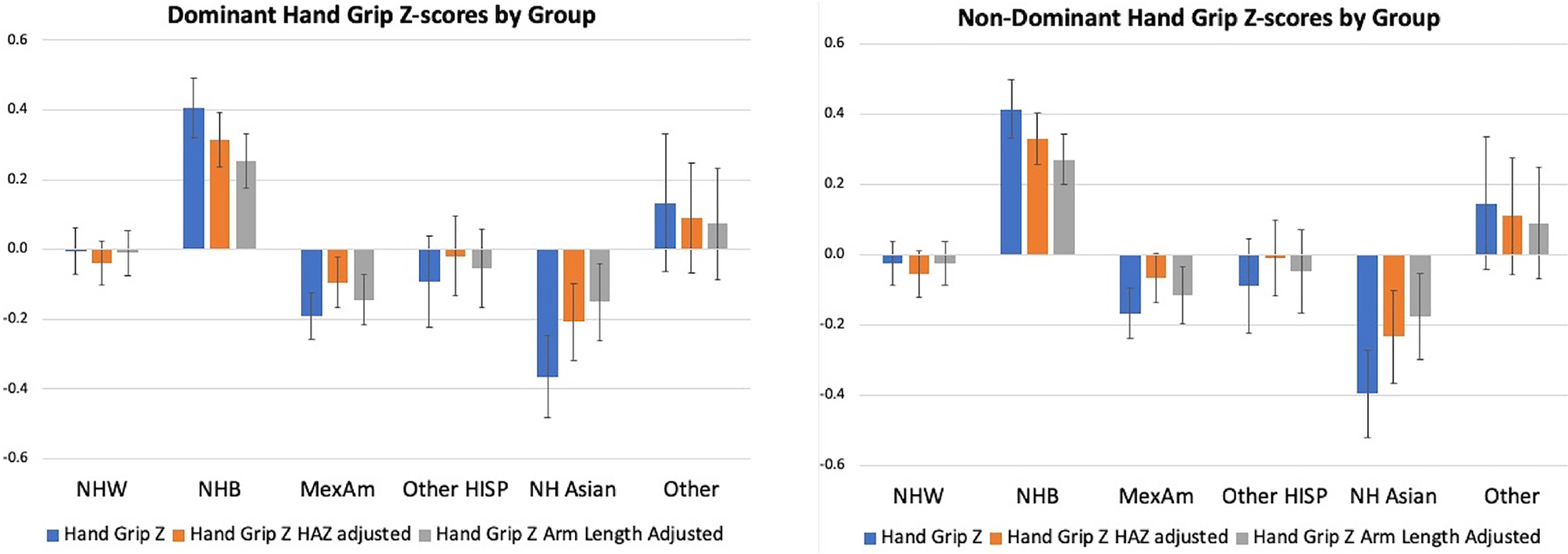
Dominant and non-dominant handgrip Z-scores by race/ethnicity group.

There were significant differences in handgrip Z-scores across gender and self-identified race/ethnicity groups. Compared to the non-Hispanic White group, the non-Hispanic Black group had significantly higher ND-HGZ and D-HGZ (*p* < .001 for both) while the Mexican American group had lower ND-HGZ and D-HGZ (*p* = .059 for ND-HGZ; *p* = .020 for D-HGZ) (Figure 3). These group differences were attenuated when HAZ-adjusted and UALZ-adjusted ND-HGZ and D-HGZ were compared as shown in Figure 3. When the interaction between gender and self-identified race/ethnicity group was tested, the non-Hispanic Black female group had higher D-HGZ (*p* = .012) and ND-HGZ (*p* = .061) compared to other groups. However, using HAZ-adjusted handgrip Z or UALZ-adjusted handgrip Z, this sex × group interaction was no longer significant. Moreover, in multivariate models, group differences in HAZ-adjusted ND-HGZ and D-HGZ were not significant when ALSTMIZ was included in the models, suggesting that group differences in handgrip strength are related to body size and composition.

## Discussion

We generated nationally representative, smoothed, age- and gender-specific reference curves and percentiles for dominant and non-dominant handgrip strength in participants ages 6–20 years using NHANES data. These are the first smoothed percentile curves that can be used to calculate individual paediatric handgrip Z-scores. Handgrip Z-scores were associated with anthropometric and body composition parameters, strengthening the utility of handgrip strength as an accessible measurement of functional body composition. This study is a significant improvement over prior studies by its robust sample size drawn from two NHANES survey periods, consideration of hand dominance, incorporation of body size and composition data, and generation of smoothed percentile curves using sample weights to produce estimates representative of the U.S. population from which exact Z-scores can be calculated. These reference data will facilitate more accurate interpretation of handgrip strength in healthy children and those with chronic conditions at risk of muscle deficits.

Handgrip strength was consistently higher in males, similar to previous reports of gender differences in combined grip strength in the NHANES sample (Ervin et al. 2013, 2014; Perna et al. 2016; Laurson et al. 2017). The magnitude of the differences increased with age with male handgrip strength accelerating after approximately age 11. Age-related handgrip strength slowed in females after approximately age 14. These findings are consistent with prior reports of the effect of puberty on strength across multiple muscle groups in United States, European, and Australian populations (Bäckman et al. 1989; Beenakker et al. 2001; Eek et al. 2006; Catley and Tomkinson 2013; Ervin et al. 2014; Perna et al. 2016). However, while dominant and non-dominant handgrip strength both increased with age in males and females, the difference between hands increased with age and became increasingly variable, supporting our description of Z-scores by hand dominance, rather than combined handgrip strength as in prior studies.

We acknowledge that self-identified race and ethnicity are social constructs, yet embedded in these constructs are factors that may contribute to growth, maturation, body composition and muscle strength, including lifestyle differences, access to resources, and population ancestry. Therefore, we examined handgrip strength across self-identified race/ethnicity groups to provide a frame of reference for future studies and clinical evaluations that use these reference ranges. Importantly, the NHANES sample is based on a multi-stage probability sampling approach to reflect the United States demographics. Like McGrath et al. (2020), we found significant differences between groups. Handgrip strength was highest in the group that self-identified as Black and lowest in the Mexican American group. D-HGZ was greatest in the non-Hispanic Black female group. However, use of size adjusted HGZ attenuated these group differences, and statistical models that adjusted for ALSTMIZ eliminated group differences. These findings demonstrate the impact of size and body composition on handgrip strength and emphasise that group differences are related to body size and composition characteristics that also vary between self-identified racial/ethnicity groups. This is useful information for interpreting handgrip Z-scores in population studies or clinical evaluations when body composition data are not available.

Handgrip strength was associated with body size, and we present a method for determining the degree to which a handgrip Z-score deviates from the expected values based on HAZ or UALZ. Height is a readily available measure in both research and clinical settings. It is a proxy measure of long bone length which is biomechanically relevant to strength measures. In children, height and age covary, yet for any given height, the age range can be fairly large (e.g. a height of 144 cm encompasses children who are at the 95th percentile at 9 years and at the 5th percentile at age 13 y). Consequently, height-based reference ranges may conflate prepubertal and pubertal children since they do not account for age. Therefore, we used height-for-age *Z*-score to account for the association of variability in body size on handgrip strength at any given age. This approach has the added advantage of partially accounting for maturational timing, since early maturing children will be taller than average at younger ages. Indeed, the attenuation of group differences in handgrip strength when HAZ-adjusted *Z*-scores were used is consistent with group differences both in size and maturational timing. Of note, we also presented body size-adjusted handgrip Z-score using UALZ with the expectation that a more specific and proximal measure of long bone length would be better than HAZ in accounting for group differences in handgrip strength, given the expected differences in body proportions between racial/ethnicity groups.

Muscle size is a major determinant of strength outcomes. We examined the association of LBMIZ and ALSTMIZ with handgrip Z-score. LBMIZ includes all lean tissues in the body including organs, whereas ALSTMIZ is based on the skeletal muscle in the arms and legs, which should be more relevant to a muscle strength outcome. Indeed, we found that the correlation coefficients with handgrip Z were higher for ALSTMIZ compared to LBMIZ. In addition, when ALSTMIZ was included in multivariate models, the differences in handgrip Z-scores among self-identified race/ethnicity group were not significant. In contrast, the association of FMIZ with handgrip Z was very low. These findings suggest that handgrip could be a surrogate for metabolically active skeletal muscle and a representation of functional body composition. While prior studies associated handgrip strength with body size (height, weight, BMI) (Laurson et al. 2017; Kocher et al. 2019; McGrath et al. 2020), weight and BMI may conceal differences in fat and lean mass distribution in certain self-identified race/ethnicity groups (Harsha et al. 1978; Schutte et al. 1984; Goran et al. 1997; Nelson and Barondess 1997; Lu et al. 2011; Sousa et al. 2013; Weber et al. 2013; Marwaha et al. 2014) and in chronic disease populations. Associations between handgrip strength and lean mass may be important in assessment of paediatric populations in which lean mass deficits (surrogates for skeletal muscle deficits) are associated with functional limitations (Avitabile et al. 2014, 2018, 2021, 2022). Future studies may explore handgrip Z-score as a marker of functional health status and potential clinical trial target in children with chronic disease.

## Limitations

There are some important limitations to these results. The NHANES data are cross-sectional, preventing us from assessing changes in handgrip with growth or lean body mass accrual. Additionally, the body composition analyses were only performed on the subset of patients with both handgrip and DXA data; however, this is the largest nationally representative sample of children with these data and provide useful reference ranges. Finally, we could not assess the effect of puberty or nutritional status on handgrip,both important influences.

In conclusion, we have generated age- and gender-specific reference curves and smoothed percentiles for dominant and non-dominant handgrip strength in NHANES participants ages 6–20 years. Handgrip Z-scores differed among self-identified racial/ethnicity groups and were associated with body size and body composition. Generating individual level Z-scores from these data will be helpful to clinicians and researchers studying handgrip strength in healthy children and those with chronic conditions that threaten muscle health.

## Supplementary Material

Supplemental Table 1

Supplemental Table 2

Supplemental Table 3

## Figures and Tables

**Table 1.** Descriptive characteristics of the NHANES sample population used to create handgrip reference curves.

Variable	*n* or %	Mean ± SD

Age, y	4290	13.1 ± 3.9
Gender^1^		
Male	50.3	
Female	49.7	
Race/Ethnicity group^1^		
Non-Hispanic White	54.9	
Non-Hispanic Black	13.9	
Mexican American	15.2	
other Hispanic	7.0	
other	9.0	
Weight, *kg*	4263	53.2 ± 23.2
WAZ	4263	0.56 ± 1.16
Height, *cm*	4264	153.2 ± 18.5
HAZ	4264	0.17 ± 1.01
BMI, *kg/m^2^*	4263	21.7 ± 6.1
BMIZ	4263	0.53 ± 1.15
upper arm length, *cm*	4202	33.0 ± 4.8
ualz	4202	0.04 ± 0.98
LBMI, *kg/m^2^*	3244	15.6 ± 3.2
LBMIZ	3244	0.25 ± 1.41
ALsTMI *kg/m^2^*	3239	6.9 ± 1.7
alstmiz	3239	0.17 ± 1.31
FMI, *kg/m^2^*	3219	6.2 ± 3.6
FMIZ	3219	0.42 ± 1.20
Dominant HG, *kg*	4290	25.9 ± 11.8
Dominant HGZ	4290	0.00 ± 0.98
Non-dominant HG, *kg*	4290	24.4 ± 11.2
Non-dominant HGZ	4290	0.01 ± 0.98

y, year; kg, kilogram; WAZ, weight Z-score; cm, centimetre; HAZ, height Z-score; BMI/Z, body mass index/Z-score; m^2^, square metre; UALZ, upper arm length Z-score; LBMI/Z, lean body mass index/Z-score; ALSTMI/Z, appendicular lean soft tissue mass index/Z-score; FMI/Z, fat mass index/Z-score; HG/HGZ, handgrip/Z-score.

1 United States population weighted proportions of gender and self-identified race/ethnicity groups.

**Table 2.** Reference tables for dominant hand grip (A), non-dominant handgrip (B), and upper arm length (C) in male participants ages 6–19.9 years.

(A) Dominant handgrip, kg									

Males									

Age, y	L	S	5th	10th	25th	50th (M)	75th	90th	95th

6.0 to 6.9	0.19	0.23	7.38	8.06	9.30	10.85	12.61	14.38	15.53
7.0 to 7.9	0.23	0.22	8.52	9.29	10.71	12.47	14.45	16.44	17.72
8.0 to 8.9	0.27	0.22	9.74	10.62	12.23	14.21	16.42	18.62	20.03
9.0 to 9.9	0.30	0.22	11.17	12.18	14.01	16.26	18.74	21.20	22.77
10.0 to 10.9	0.33	0.21	12.90	14.06	16.17	18.73	21.55	24.33	26.10
11.0 to 11.9	0.35	0.21	15.02	16.36	18.79	21.74	24.98	28.15	30.17
12.0 to 12.9	0.37	0.21	17.59	19.15	21.96	25.36	29.08	32.71	35.01
13.0 to 13.9	0.38	0.20	20.54	22.33	25.55	29.43	33.65	37.77	40.37
14.0 to 14.9	0.40	0.20	23.59	25.60	29.20	33.51	38.19	42.73	45.59
15.0 to 15.9	0.42	0.19	26.50	28.68	32.57	37.23	42.24	47.08	50.12
16.0 to 16.9	0.43	0.19	29.09	31.39	35.48	40.34	45.56	50.59	53.73
17.0 to 17.9	0.44	0.18	31.34	33.71	37.90	42.87	48.19	53.29	56.48
18.0 to 18.9	0.44	0.17	33.33	35.74	39.98	44.99	50.35	55.47	58.66
19.0 to 19.9	0.44	0.16	35.20	37.63	41.90	46.94	52.30	57.41	60.59

(B) Non-dominant handgrip, kg									

Males									

Age, y	L	S	5th	10th	25th	50th (M)	75th	90th	95th

6.0 to 6.9	0.24	0.23	7.05	7.71	8.94	10.46	12.17	13.88	14.99
7.0 to 7.9	0.25	0.23	8.10	8.86	10.25	11.97	13.90	15.84	17.09
8.0 to 8.9	0.27	0.22	9.24	10.09	11.66	13.59	15.75	17.91	19.29
9.0 to 9.9	0.29	0.22	10.57	11.55	13.32	15.51	17.93	20.34	21.88
10.0 to 10.9	0.32	0.22	12.20	13.31	15.34	17.83	20.57	23.28	25.01
11.0 to 11.9	0.35	0.21	14.18	15.48	17.82	20.67	23.80	26.88	28.84
12.0 to 12.9	0.37	0.21	16.55	18.05	20.76	24.04	27.63	31.14	33.37
13.0 to 13.9	0.40	0.21	19.23	20.96	24.06	27.80	31.88	35.85	38.35
14.0 to 14.9	0.43	0.20	22.00	23.95	27.42	31.60	36.11	40.48	43.23
15.0 to 15.9	0.45	0.20	24.63	26.76	30.55	35.06	39.92	44.60	47.52
16.0 to 16.9	0.49	0.19	26.96	29.23	33.24	38.00	43.09	47.95	50.99
17.0 to 17.9	0.53	0.19	28.97	31.35	35.53	40.45	45.66	50.62	53.69
18.0 to 18.9	0.59	0.18	30.74	33.21	37.53	42.57	47.86	52.85	55.93
19.0 to 19.9	0.65	0.17	32.41	34.97	39.41	44.55	49.90	54.91	57.98

(C) upper arm length, cm									

Age, y	L	S	5th	10th	25th	50th (M)	75th	90th	95th

6.0 to 6.9	0.23	0.07	22.32	22.87	23.82	24.90	26.02	27.06	27.70
7.0 tp 7.9	0.27	0.07	23.60	24.19	25.20	26.36	27.56	28.67	29.35
8.0 to 8.9	0.29	0.07	24.88	25.51	26.59	27.82	29.10	30.28	31.00
9.0 to 9.9	0.25	0.07	26.20	26.86	28.00	29.31	30.66	31.92	32.69
10.0 to 10.9	0.15	0.07	27.56	28.26	29.46	30.83	32.26	33.60	34.43
11.0 to 11.9	0.00	0.07	28.96	29.68	30.92	32.36	33.86	35.28	36.16
12.0 to 12.9	−0.15	0.07	30.31	31.05	32.33	33.81	35.38	36.87	37.79
13.0 to 13.9	−0.28	0.07	31.55	32.30	33.60	35.13	36.74	38.28	39.24
14.0 to 14.9	−0.38	0.07	32.61	33.37	34.68	36.23	37.87	39.44	40.42
15.0 to 15.9	−0.40	0.06	33.45	34.21	35.53	37.08	38.73	40.31	41.30
16.0 to 16.9	−0.35	0.06	34.06	34.82	36.15	37.70	39.35	40.92	41.89
17.0 to 17.9	−0.23	0.06	34.48	35.25	36.58	38.13	39.76	41.31	42.27
18.0 to 18.9	−0.06	0.06	34.78	35.56	36.89	38.44	40.05	41.57	42.50
19.0 to 19.9	0.13	0.06	35.03	35.81	37.16	38.70	40.29	41.78	42.69

**Table 3.** Reference tables for dominant hand grip (A), non-dominant handgrip (B), and upper arm length (C) in female participants ages 6–19.9 years.(A) Dominant handgrip, kg

Females									
	
Age, y	L	S	5th	10th	25th	50th (M)	75th	90th	95th

6.0 to 6.9	−0.28	0.22	7.05	7.58	8.58	9.89	11.46	13.18	14.36
7.0 to 7.9	−0.16	0.21	8.33	8.97	10.18	11.74	13.58	15.54	16.87
8.0 to 8.9	−0.06	0.21	9.72	10.49	11.92	13.75	15.88	18.11	19.60
9.0 to 9.9	0.02	0.21	11.24	12.15	13.83	15.95	18.40	20.92	22.58
10.0 to 10.9	0.08	0.21	12.88	13.93	15.86	18.29	21.05	23.87	25.71
11.0 to 11.9	0.13	0.21	14.56	15.75	17.92	20.63	23.69	26.78	28.79
12.0 to 12.9	0.16	0.20	16.20	17.51	19.88	22.83	26.13	29.45	31.59
13.0 to 13.9	0.18	0.20	17.72	19.11	21.64	24.75	28.23	31.70	33.93
14.0 to 14.9	0.21	0.19	19.03	20.49	23.12	26.35	29.92	33.46	35.73
15.0 to 15.9	0.25	0.19	20.10	21.61	24.31	27.60	31.22	34.77	37.04
16.0 to 16.9	0.31	0.18	20.93	22.48	25.25	28.58	32.20	35.73	37.95
17.0 to 17.9	0.38	0.18	21.60	23.18	26.00	29.36	32.97	36.45	38.63
18.0 to 18.9	0.46	0.17	22.17	23.80	26.66	30.03	33.62	37.04	39.18
19.0 to 19.9	0.54	0.17	22.73	24.39	27.28	30.67	34.23	37.60	39.68

(B) Non-dominant handgrip, kg									

Females									

Age, y	L	S	5th	10th	25th	50th (M)	75th	90th	95th

6.0 to 6.9	−0.43	0.22	6.86	7.35	8.30	9.58	11.15	12.91	14.15
7.0 to 7.9	−0.27	0.22	8.01	8.62	9.77	11.28	13.10	15.08	16.44
8.0 to 8.9	−0.12	0.22	9.23	9.96	11.33	13.10	15.19	17.40	18.88
9.0 to 9.9	0.00	0.22	10.56	11.43	13.04	15.09	17.46	19.92	21.55
10.0 to 10.9	0.10	0.22	11.98	12.99	14.85	17.19	19.86	22.58	24.36
11.0 to 11.9	0.17	0.21	13.43	14.58	16.68	19.30	22.25	25.22	27.14
12.0 to 12.9	0.22	0.21	14.85	16.12	18.43	21.28	24.47	27.65	29.70
13.0 to 13.9	0.25	0.21	16.17	17.52	19.99	23.02	26.37	29.70	31.83
14.0 to 14.9	0.28	0.20	17.31	18.73	21.31	24.45	27.91	31.32	33.49
15.0 to 15.9	0.32	0.19	18.24	19.72	22.37	25.58	29.10	32.53	34.71
16.0 to 16.9	0.37	0.19	18.97	20.49	23.21	26.47	30.01	33.44	35.60
17.0 to 17.9	0.44	0.19	19.54	21.11	23.88	27.19	30.73	34.15	36.28
18.0 to 18.9	0.50	0.18	20.01	21.62	24.45	27.79	31.34	34.73	36.83
19.0 to 19.9	0.57	0.18	20.44	22.09	24.98	28.35	31.90	35.26	37.34

(C) upper arm length, cm									

Age, y	L	S	5th	10th	25th	50th (M)	75th	90th	95th

6.0 to 6.9	−0.82	0.07	22.11	22.63	23.55	24.64	25.84	27.01	27.75
7.0 to 7.9	−0.82	0.07	23.53	24.08	25.06	26.22	27.49	28.73	29.52
8.0 to 8.9	−0.79	0.07	24.95	25.54	26.57	27.80	29.14	30.45	31.28
9.0 to 9.9	−0.72	0.07	26.35	26.96	28.05	29.35	30.75	32.12	32.99
10.0 to 10.9	−0.59	0.07	27.65	28.30	29.45	30.80	32.26	33.67	34.56
11.0 to 11.9	−0.42	0.07	28.82	29.50	30.70	32.10	33.60	35.03	35.93
12.0 to 12.9	−0.23	0.07	29.81	30.52	31.75	33.19	34.70	36.14	37.04
13.0 to 13.9	−0.04	0.07	30.58	31.31	32.56	34.02	35.55	36.98	37.87
14.0 to 14.9	0.14	0.06	31.12	31.86	33.14	34.61	36.14	37.57	38.44
15.0 to 15.9	0.31	0.06	31.47	32.22	33.52	35.00	36.52	37.94	38.80
16.0 to 16.9	0.46	0.06	31.68	32.45	33.76	35.24	36.76	38.16	39.01
17.0 to 17.9	0.61	0.06	31.82	32.60	33.92	35.41	36.92	38.31	39.15
18.0 to 18.9	0.76	0.06	31.92	32.71	34.05	35.54	37.05	38.43	39.26
19.0 to 19.9	0.91	0.06	32.02	32.82	34.17	35.67	37.17	38.54	39.35

**Table 4.** Correlations between unadjusted and adjusted handgrip Z-scores, anthropometric, and body composition measures using data from 3209 NHANEs participants with complete DXA data.

	HAZ	BMIZ	UALZ	LBMIZ	ALSTMIZ	FMIZ

Non-dominant HGZ	0.42 *p* < .001	0.36 *p* < .001	0.46 *p* < .001	0.51 *p* < .001	0.54 *p* < .001	0.17 *p* < .001
Non-dominant HGZ, adjusted for HAZ	−0.02 *p* = .46	0.30 *p* < .001	0.11 *p* < .001	0.43 *p* < .001	0.46 *p* < .001	0.12 *p* < .001
Non-dominant HGZ, adjusted for UALZ	0.11 *p* < .001	0.16 *p* < .001	−0.02 *p* = .33	0.31 *p* < .001	0.34 *p* < .001	−0.01 *p* = .62
Dominant HGZ	0.43 *p* < .001	0.37 *p* < .001	0.47 *p* < .001	0.51 *p* < .001	0.54 *p* < .001	0.17 *p* < .001
Dominant HGZ, adjusted for HAZ	−0.01 *p* = .64	0.31 *p* < .001	0.12 *p* < .001	0.44 *p* < .001	0.46 *p* < .001	0.12 *p* < .001
Dominant HGZ, adjusted for uALZ	0.13 *p* < .001	0.17 *p* < .001	−0.02 *p* = .46	0.30 *p* < .001	0.34 *p* < .001	−0.02 *p* = .47

HAZ, height Z-score; BMIZ, body mass index Z-score; UALZ, upper arm length Z-score; LBMIZ, lean body mass index Z-score; ALSTMIZ, appendicular lean soft tissue mass index Z-score; FMIZ, fat mass index Z-score. HGZ, handgrip Z-score.

**Table 5.** Equations to predict D-HGZ and ND-HGZ based on body size as described in Equation 3 in text.

Dominant hand grip, kg				

	HAZ adjustment equations		UALZ adjustment equations	
		
Age, y	Males (*R*^2^ = 0.28, *n* = 2142)	Females (*R*^2^ = 0.24, *n* = 2122)	Males (*R*^2^ = 0.25, *n* = 2113)	Females (*R*^2^ = 0.26, *n* = 2089)

6.0 to 6.9	−0.223 + (HAZ * 0.356)	−0.087 + (HAZ * 0.476)	−0.077 + (UALZ * 0.344)	0.035 + (UALZ * 0.395)
7.0 to 7.9	−0.109 + (HAZ * 0.391)	−0.199 + (HAZ * 0.460)	−0.005 + (UALZ * 0.454)	−0.152 + (UALZ * 0.445)
8.0 to 8.9	−0.125 + (HAZ * 0.457)	−0.286 + (HAZ * 0.423)	0.014 + (UALZ * 0.458)	−0.143 + (UALZ * 0.392)
9.0 to 9.9	−0.120 + (HAZ * 0.405)	−0.421 + (HAZ * 0.534)	−0.011 + (UALZ * 0.399)	−0.178 + (UALZ * 0.479)
10.0 to 10.9	−0.248 + (HAZ * 0.619)	−0.413 + (HAZ * 0.635)	0.078 + (UALZ * 0.553)	−0.107 + (UALZ * 0.532)
11.0 to 11.9	−0.430 + (HAZ * 0.604)	−0.341 + (HAZ * 0.602)	−0.093 + (UALZ * 0.635)	−0.115 + (UALZ * 0.618)
12.0 to 12.9	−0.588 + (HAZ * 0.627)	0.039 + (HAZ * 0.585)	−0.239 + (UALZ * 0.556)	0.054 + (UALZ * 0.685)
13.0 to 13.9	−0.321 + (HAZ * 0.745)	0.035 + (HAZ * 0.435)	−0.091 + (UALZ * 0.610)	−0.051 + (UALZ * 0.434)
14.0 to 14.9	−0.055 + (HAZ * 0.537)	0.283 + (HAZ * 0.295)	0.052 + (UALZ * 0.464)	0.191 + (UALZ * 0.357)
15.0 to 15.9	0.193 + (HAZ * 0.441)	0.238 + (HAZ * 0.334)	0.219 + (UALZ * 0.473)	0.147 + (UALZ * 0.303)
16.0 to 16.9	0.285 + (HAZ * 0.330)	−0.044 + (HAZ * 0.261)	0.221 + (UALZ * 0.279)	−0.055 + (UALZ * 0.325)
17.0 to 17.9	0.065 + (HAZ * 0.298)	0.184 + (HAZ * 0.459)	0.023 + (UALZ * 0.293)	0.074 + (UALZ * 0.620)
18.0 to 18.9	0.016 + (HAZ * 0.340)	0.016 + (HAZ * 0.464)	−0.021 + (UALZ * 0.494)	−0.048 + (UALZ * 0.473)
19.0 to 19.9	−0.123 + (HAZ * 0.562)	0.113 + (HAZ * 0.401)	−0.054 + (HAZ * 0.694)	0.033 + (HAZ * 0.367)

Non-dominant hand grip, kg				

	HAZ adjustment equations		UALZ adjustment equations	
		
Age, y	Males (*R*^2^ = 0.26, *n* = 2142)	Females (*R*^2^ = 0.23, *n* = 2122)	Males (*R*^2^ = 0.23, *n* = 2113)	Females (*R*^2^ = 0.26, *n* = 2089)

6.0 to 6.9	−0.202 + (HAZ * 0.394)	−0.147 + (HAZ * 0.540)	−0.039 + (UALZ * 0.390)	−0.005 + (UALZ * 0.463)
7.0 tp 7.9	−0.135 + (HAZ * 0.367)	−0.216 + (HAZ * 0.464)	−0.037 + (UALZ * 0.424)	−0.157 + (UALZ * 0.441)
8.0 to 8.9	−0.124 + (HAZ * 0.489)	−0.305 + (HAZ * 0.372)	0.026 + (UALZ * 0.461)	−0.175 + (UALZ * 0.390)
9.0 to 9.9	−0.115 + (HAZ * 0.382)	−0.466 + (HAZ * 0.582)	−0.018 + (UALZ * 0.348)	−0.203 + (UALZ * 0.533)
10.0 to 10.9	−0.291 + (HAZ * 0.582)	−0.360 + (HAZ * 0.592)	0.021 + (UALZ * 0.541)	−0.070 + (UALZ * 0.478)
11.0 to 11.9	−0.310 + (HAZ * 0.523)	−0.307 + (HAZ * 0.559)	−0.027 + (UALZ * 0.557)	−0.097 + (UALZ * 0.565)
12.0 to 12.9	−0.616 + (HAZ * 0.634)	0.094 + (HAZ * 0.547)	−0.264 + (UALZ * 0.561)	0.106 + (UALZ * 0.646)
13.0 to 13.9	−0.356 + (HAZ * 0.724)	0.041 + (HAZ * 0.395)	−0.141 + (UALZ * 0.618)	−0.042 + (UALZ * 0.435)
14.0 to 14.9	−0.023 + (HAZ * 0.466)	0.248 + (HAZ * 0.331)	0.072 + (UALZ * 0.414)	0.133 + (UALZ * 0.507)
15.0 to 15.9	0.211 + (HAZ * 0.400)	0.174 + (HAZ * 0.294)	0.228 + (UALZ * 0.458)	0.084 + (UALZ * 0.277)
16.0 to 16.9	0.333 + (HAZ * 0.494)	−0.046 + (HAZ * 0.280)	0.221 + (UALZ * 0.358)	−0.056 + (UALZ * 0.384)
17.0 to 17.9	−0.023 + (HAZ * 0.295)	0.189 + (HAZ * 0.500)	−0.066 + (UALZ * 0.260)	0.074 + (UALZ * 0.579)
18.0 to 18.9	0.075 + (HAZ * 0.268)	0.083 + (HAZ * 0.448)	0.044 + (UALZ * 0.467)	0.021 + (UALZ * 0.463)
19.0 to 19.9	−0.118 + (HAZ * 0.520)	0.019 + (HAZ * 0.452)	−0.064 + (UALZ * 0.583)	−0.065 + (UALZ * 0.375)

HAZ, Height for Age Z-score; UALZ, upper Arm Length Z-score.

## Data Availability

The data that support the findings of this study are openly available from the Centres for Disease Control and Prevention (CDC), National Centre for Health Statistics (NCHS). National Health and Nutrition Examination Survey Data. Hyattsville, MD: U.S. Department of Health and Human Services, Centres for Disease Control and Prevention, 2011–2014, https://wwwn.cdc.gov/nchs/nhanes/continuousnhanes/overview.aspx?BeginYear=2011 and https://wwwn.cdc.gov/nchs/nhanes/continuousnhanes/overview.aspx?BeginYear=2013.
