# Supplementary material for: Paediatric dominant and non-dominant handgrip reference curves and the association with body composition
Source: Ann Hum Biol. Author manuscript; Available in PMC 2024 Jun 10. (PMC11164034; doi:10.1080/03014460.2023.2298474)
Supplement: Supplemental Table 1 [file NIHMS1991062-supplement-Supplemental_Table_1.docx]

**Supplemental Table 1. Descriptive characteristics of the NHANES sample population with both handgrip and DXA data**

| **Variable** | **n or %** | **Mean±SD** |
| --- | --- | --- |
| Age, *y* | 3,209 | 13.9±3.4 |
| Gender^1^ |  |  |
| Male | 50.0 |  |
| Female | 50.0 |  |
| Ancestry group^1^ |  |  |
| Non-Hispanic White | 56.2 |  |
| Non-Hispanic Black | 13.5 |  |
| Mexican American | 15.1 |  |
| Other Hispanic | 6.8 |  |
| Other | 8.4 |  |
| Weight, *kg* | 3,209 | 57.1±21.4 |
| WAZ | 3,209 | 0.57±1.15 |
| Height, *cm* | 3,209 | 157.6±14.8 |
| HAZ | 3,209 | 0.14±1.01 |
| BMI, *kg/m^2^* | 3,209 | 22.4±6.0 |
| BMIZ | 3,209 | 0.55±1.14 |
| Upper arm length, *cm* | 3,209 | 34.1±3.9 |
| UALZ | 3,209 | 0.03±0.97 |
| LBMI, *kg/m^2^* | 3,209 | 15.6±3.2 |
| LBMIZ | 3,209 | 0.25±1.41 |
| ALSTMI *kg/m^2^* | 3,209 | 6.9±1.7 |
| ALSTMIZ | 3,209 | 0.17±1.31 |
| FMI, *kg/m^2^* | 3,209 | 6.2±3.6 |
| FMIZ | 3,209 | 0.42±1.20 |
| Dominant HG, *kg* | 3,209 | 27.9±10.9 |
| Dominant HGZ | 3,209 | 0.00±0.97 |
| Non-dominant HG, *kg* | 3,209 | 26.3±10.4 |
| Non-dominant HGZ | 3,209 | 0.00±0.98 |

y, year; kg, kilogram; WAZ, weight Z-score; cm, centimeter; HAZ, height Z-score; BMI/Z, body mass index/Z-score; m^2^, square meter; UALZ, upper arm length/Z-score; LBMI/Z, lean body mass index/Z-score; ALSTMI/Z, appendicular lean soft tissue mass index/Z-score; FMI/Z, fat mass index/Z-score; HG/HGZ, handgrip/Z-score

^1^Weighted estimate
