# Supplementary material for: Paediatric dominant and non-dominant handgrip reference curves and the association with body composition
Source: Ann Hum Biol. Author manuscript; Available in PMC 2024 Jun 10. (PMC11164034; doi:10.1080/03014460.2023.2298474)
Supplement: Supplemental Table 2 [file NIHMS1991062-supplement-Supplemental_Table_2.docx]

**Supplemental Table 2 Reference tables for dominant hand grip (A), non-dominant handgrip (B), and upper arm length (C) in males ages 6-19.9 years. Age in tenths of a year.**

1. Dominant handgrip, kg

|  | Males | | | | | | | | |
| --- | --- | --- | --- | --- | --- | --- | --- | --- | --- |
| Age, y | L | S | 5th | 10th | 25th | 50th (M) | 75th | 90th | 95th |
| 6.0 | 0.18 | 0.23 | 6.82 | 7.45 | 8.60 | 10.05 | 11.69 | 13.36 | 14.44 |
| 6.1 | 0.18 | 0.23 | 6.94 | 7.57 | 8.74 | 10.21 | 11.87 | 13.56 | 14.66 |
| 6.2 | 0.18 | 0.23 | 7.05 | 7.69 | 8.88 | 10.37 | 12.06 | 13.77 | 14.88 |
| 6.3 | 0.19 | 0.23 | 7.16 | 7.82 | 9.02 | 10.53 | 12.24 | 13.97 | 15.09 |
| 6.4 | 0.19 | 0.23 | 7.27 | 7.94 | 9.16 | 10.69 | 12.42 | 14.18 | 15.31 |
| 6.5 | 0.19 | 0.23 | 7.38 | 8.06 | 9.30 | 10.85 | 12.61 | 14.38 | 15.53 |
| 6.6 | 0.20 | 0.23 | 7.50 | 8.18 | 9.44 | 11.01 | 12.79 | 14.59 | 15.75 |
| 6.7 | 0.20 | 0.22 | 7.61 | 8.30 | 9.58 | 11.18 | 12.97 | 14.79 | 15.97 |
| 6.8 | 0.20 | 0.22 | 7.72 | 8.43 | 9.72 | 11.34 | 13.16 | 14.99 | 16.19 |
| 6.9 | 0.21 | 0.22 | 7.83 | 8.55 | 9.86 | 11.50 | 13.34 | 15.20 | 16.40 |
| 7.0 | 0.21 | 0.22 | 7.95 | 8.67 | 10.00 | 11.66 | 13.52 | 15.40 | 16.62 |
| 7.1 | 0.21 | 0.22 | 8.06 | 8.80 | 10.14 | 11.82 | 13.71 | 15.61 | 16.84 |
| 7.2 | 0.22 | 0.22 | 8.18 | 8.92 | 10.28 | 11.98 | 13.89 | 15.81 | 17.06 |
| 7.3 | 0.22 | 0.22 | 8.29 | 9.04 | 10.43 | 12.15 | 14.08 | 16.02 | 17.28 |
| 7.4 | 0.22 | 0.22 | 8.40 | 9.17 | 10.57 | 12.31 | 14.26 | 16.23 | 17.50 |
| 7.5 | 0.23 | 0.22 | 8.52 | 9.29 | 10.71 | 12.47 | 14.45 | 16.44 | 17.72 |
| 7.6 | 0.23 | 0.22 | 8.64 | 9.42 | 10.86 | 12.64 | 14.64 | 16.64 | 17.94 |
| 7.7 | 0.24 | 0.22 | 8.75 | 9.55 | 11.00 | 12.81 | 14.83 | 16.85 | 18.16 |
| 7.8 | 0.24 | 0.22 | 8.87 | 9.68 | 11.15 | 12.98 | 15.02 | 17.07 | 18.39 |
| 7.9 | 0.24 | 0.22 | 8.99 | 9.81 | 11.30 | 13.15 | 15.21 | 17.28 | 18.61 |
| 8.0 | 0.25 | 0.22 | 9.11 | 9.94 | 11.45 | 13.32 | 15.41 | 17.49 | 18.84 |
| 8.1 | 0.25 | 0.22 | 9.23 | 10.07 | 11.60 | 13.49 | 15.60 | 17.71 | 19.07 |
| 8.2 | 0.26 | 0.22 | 9.35 | 10.20 | 11.75 | 13.66 | 15.80 | 17.93 | 19.30 |
| 8.3 | 0.26 | 0.22 | 9.48 | 10.34 | 11.91 | 13.84 | 16.00 | 18.16 | 19.54 |
| 8.4 | 0.26 | 0.22 | 9.61 | 10.48 | 12.07 | 14.02 | 16.21 | 18.38 | 19.78 |
| 8.5 | 0.27 | 0.22 | 9.74 | 10.62 | 12.23 | 14.21 | 16.42 | 18.62 | 20.03 |
| 8.6 | 0.27 | 0.22 | 9.87 | 10.76 | 12.39 | 14.40 | 16.63 | 18.85 | 20.28 |
| 8.7 | 0.28 | 0.22 | 10.00 | 10.91 | 12.56 | 14.59 | 16.85 | 19.09 | 20.53 |
| 8.8 | 0.28 | 0.22 | 10.14 | 11.06 | 12.73 | 14.78 | 17.07 | 19.34 | 20.79 |
| 8.9 | 0.28 | 0.22 | 10.28 | 11.21 | 12.90 | 14.98 | 17.29 | 19.59 | 21.06 |
| 9.0 | 0.29 | 0.22 | 10.42 | 11.36 | 13.08 | 15.18 | 17.52 | 19.84 | 21.33 |
| 9.1 | 0.29 | 0.22 | 10.56 | 11.52 | 13.26 | 15.39 | 17.76 | 20.11 | 21.61 |
| 9.2 | 0.29 | 0.22 | 10.71 | 11.68 | 13.44 | 15.60 | 18.00 | 20.37 | 21.89 |
| 9.3 | 0.30 | 0.22 | 10.86 | 11.85 | 13.63 | 15.82 | 18.24 | 20.64 | 22.18 |
| 9.4 | 0.30 | 0.22 | 11.02 | 12.01 | 13.82 | 16.04 | 18.49 | 20.92 | 22.47 |
| 9.5 | 0.30 | 0.22 | 11.17 | 12.18 | 14.01 | 16.26 | 18.74 | 21.20 | 22.77 |
| 9.6 | 0.31 | 0.22 | 11.33 | 12.36 | 14.21 | 16.49 | 19.00 | 21.49 | 23.08 |
| 9.7 | 0.31 | 0.22 | 11.49 | 12.53 | 14.41 | 16.72 | 19.27 | 21.78 | 23.39 |
| 9.8 | 0.31 | 0.21 | 11.66 | 12.71 | 14.62 | 16.95 | 19.53 | 22.08 | 23.71 |
| 9.9 | 0.32 | 0.21 | 11.83 | 12.89 | 14.83 | 17.19 | 19.81 | 22.38 | 24.03 |
| 10.0 | 0.32 | 0.21 | 12.00 | 13.08 | 15.04 | 17.44 | 20.08 | 22.69 | 24.36 |
| 10.1 | 0.32 | 0.21 | 12.17 | 13.27 | 15.26 | 17.69 | 20.37 | 23.01 | 24.69 |
| 10.2 | 0.32 | 0.21 | 12.35 | 13.46 | 15.48 | 17.94 | 20.65 | 23.33 | 25.03 |
| 10.3 | 0.33 | 0.21 | 12.53 | 13.66 | 15.70 | 18.20 | 20.95 | 23.66 | 25.38 |
| 10.4 | 0.33 | 0.21 | 12.71 | 13.86 | 15.93 | 18.46 | 21.25 | 23.99 | 25.74 |
| 10.5 | 0.33 | 0.21 | 12.90 | 14.06 | 16.17 | 18.73 | 21.55 | 24.33 | 26.10 |
| 10.6 | 0.33 | 0.21 | 13.09 | 14.27 | 16.41 | 19.01 | 21.87 | 24.68 | 26.47 |
| 10.7 | 0.34 | 0.21 | 13.29 | 14.49 | 16.65 | 19.28 | 22.18 | 25.04 | 26.85 |
| 10.8 | 0.34 | 0.21 | 13.49 | 14.70 | 16.90 | 19.57 | 22.51 | 25.40 | 27.24 |
| 10.9 | 0.34 | 0.21 | 13.69 | 14.93 | 17.15 | 19.86 | 22.84 | 25.77 | 27.63 |
| 11.0 | 0.34 | 0.21 | 13.90 | 15.15 | 17.41 | 20.16 | 23.18 | 26.15 | 28.03 |
| 11.1 | 0.34 | 0.21 | 14.12 | 15.39 | 17.68 | 20.46 | 23.53 | 26.53 | 28.44 |
| 11.2 | 0.35 | 0.21 | 14.34 | 15.62 | 17.95 | 20.77 | 23.88 | 26.93 | 28.86 |
| 11.3 | 0.35 | 0.21 | 14.56 | 15.87 | 18.22 | 21.09 | 24.24 | 27.33 | 29.29 |
| 11.4 | 0.35 | 0.21 | 14.79 | 16.11 | 18.50 | 21.41 | 24.61 | 27.74 | 29.72 |
| 11.5 | 0.35 | 0.21 | 15.02 | 16.36 | 18.79 | 21.74 | 24.98 | 28.15 | 30.17 |
| 11.6 | 0.35 | 0.21 | 15.25 | 16.62 | 19.08 | 22.08 | 25.36 | 28.58 | 30.62 |
| 11.7 | 0.35 | 0.21 | 15.49 | 16.88 | 19.38 | 22.42 | 25.75 | 29.01 | 31.08 |
| 11.8 | 0.36 | 0.21 | 15.74 | 17.15 | 19.68 | 22.76 | 26.14 | 29.45 | 31.54 |
| 11.9 | 0.36 | 0.21 | 15.99 | 17.42 | 19.99 | 23.12 | 26.54 | 29.89 | 32.02 |
| 12.0 | 0.36 | 0.21 | 16.24 | 17.70 | 20.31 | 23.48 | 26.95 | 30.34 | 32.50 |
| 12.1 | 0.36 | 0.21 | 16.50 | 17.98 | 20.63 | 23.84 | 27.36 | 30.81 | 32.99 |
| 12.2 | 0.36 | 0.21 | 16.77 | 18.26 | 20.95 | 24.21 | 27.78 | 31.27 | 33.48 |
| 12.3 | 0.36 | 0.21 | 17.04 | 18.55 | 21.28 | 24.59 | 28.21 | 31.75 | 33.99 |
| 12.4 | 0.36 | 0.21 | 17.31 | 18.85 | 21.62 | 24.97 | 28.64 | 32.23 | 34.50 |
| 12.5 | 0.37 | 0.21 | 17.59 | 19.15 | 21.96 | 25.36 | 29.08 | 32.71 | 35.01 |
| 12.6 | 0.37 | 0.21 | 17.87 | 19.45 | 22.30 | 25.75 | 29.52 | 33.20 | 35.54 |
| 12.7 | 0.37 | 0.21 | 18.15 | 19.76 | 22.65 | 26.15 | 29.97 | 33.70 | 36.06 |
| 12.8 | 0.37 | 0.21 | 18.44 | 20.07 | 23.00 | 26.55 | 30.42 | 34.20 | 36.60 |
| 12.9 | 0.37 | 0.21 | 18.74 | 20.39 | 23.36 | 26.95 | 30.88 | 34.71 | 37.13 |
| 13.0 | 0.37 | 0.21 | 19.03 | 20.71 | 23.72 | 27.36 | 31.34 | 35.22 | 37.67 |
| 13.1 | 0.37 | 0.21 | 19.33 | 21.03 | 24.08 | 27.77 | 31.80 | 35.73 | 38.21 |
| 13.2 | 0.38 | 0.21 | 19.63 | 21.35 | 24.44 | 28.18 | 32.26 | 36.24 | 38.75 |
| 13.3 | 0.38 | 0.21 | 19.93 | 21.68 | 24.81 | 28.60 | 32.72 | 36.75 | 39.29 |
| 13.4 | 0.38 | 0.20 | 20.23 | 22.00 | 25.18 | 29.01 | 33.19 | 37.26 | 39.83 |
| 13.5 | 0.38 | 0.20 | 20.54 | 22.33 | 25.55 | 29.43 | 33.65 | 37.77 | 40.37 |
| 13.6 | 0.38 | 0.20 | 20.84 | 22.66 | 25.91 | 29.84 | 34.12 | 38.28 | 40.91 |
| 13.7 | 0.38 | 0.20 | 21.15 | 22.99 | 26.28 | 30.26 | 34.58 | 38.79 | 41.45 |
| 13.8 | 0.39 | 0.20 | 21.46 | 23.32 | 26.65 | 30.67 | 35.04 | 39.30 | 41.98 |
| 13.9 | 0.39 | 0.20 | 21.76 | 23.65 | 27.02 | 31.08 | 35.50 | 39.80 | 42.51 |
| 14.0 | 0.39 | 0.20 | 22.07 | 23.98 | 27.38 | 31.49 | 35.96 | 40.30 | 43.03 |
| 14.1 | 0.39 | 0.20 | 22.38 | 24.30 | 27.75 | 31.90 | 36.41 | 40.79 | 43.56 |
| 14.2 | 0.39 | 0.20 | 22.68 | 24.63 | 28.12 | 32.31 | 36.86 | 41.29 | 44.07 |
| 14.3 | 0.40 | 0.20 | 22.99 | 24.96 | 28.48 | 32.71 | 37.31 | 41.77 | 44.58 |
| 14.4 | 0.40 | 0.20 | 23.29 | 25.28 | 28.84 | 33.12 | 37.75 | 42.25 | 45.09 |
| 14.5 | 0.40 | 0.20 | 23.59 | 25.60 | 29.20 | 33.51 | 38.19 | 42.73 | 45.59 |
| 14.6 | 0.40 | 0.20 | 23.89 | 25.93 | 29.55 | 33.91 | 38.62 | 43.20 | 46.08 |
| 14.7 | 0.40 | 0.20 | 24.19 | 26.24 | 29.90 | 34.30 | 39.05 | 43.66 | 46.56 |
| 14.8 | 0.41 | 0.20 | 24.49 | 26.56 | 30.25 | 34.68 | 39.47 | 44.12 | 47.04 |
| 14.9 | 0.41 | 0.20 | 24.79 | 26.87 | 30.60 | 35.06 | 39.89 | 44.57 | 47.50 |
| 15.0 | 0.41 | 0.20 | 25.08 | 27.18 | 30.94 | 35.44 | 40.30 | 45.01 | 47.96 |
| 15.1 | 0.41 | 0.20 | 25.37 | 27.49 | 31.27 | 35.81 | 40.70 | 45.44 | 48.41 |
| 15.2 | 0.41 | 0.19 | 25.65 | 27.79 | 31.61 | 36.17 | 41.10 | 45.86 | 48.85 |
| 15.3 | 0.42 | 0.19 | 25.94 | 28.09 | 31.93 | 36.53 | 41.49 | 46.28 | 49.29 |
| 15.4 | 0.42 | 0.19 | 26.22 | 28.39 | 32.26 | 36.88 | 41.87 | 46.69 | 49.71 |
| 15.5 | 0.42 | 0.19 | 26.50 | 28.68 | 32.57 | 37.23 | 42.24 | 47.08 | 50.12 |
| 15.6 | 0.42 | 0.19 | 26.77 | 28.97 | 32.89 | 37.57 | 42.61 | 47.47 | 50.53 |
| 15.7 | 0.42 | 0.19 | 27.04 | 29.26 | 33.20 | 37.90 | 42.97 | 47.86 | 50.92 |
| 15.8 | 0.42 | 0.19 | 27.31 | 29.54 | 33.50 | 38.23 | 43.32 | 48.23 | 51.30 |
| 15.9 | 0.43 | 0.19 | 27.57 | 29.82 | 33.80 | 38.55 | 43.66 | 48.59 | 51.68 |
| 16.0 | 0.43 | 0.19 | 27.84 | 30.09 | 34.09 | 38.86 | 44.00 | 48.95 | 52.04 |
| 16.1 | 0.43 | 0.19 | 28.09 | 30.36 | 34.38 | 39.17 | 44.33 | 49.29 | 52.40 |
| 16.2 | 0.43 | 0.19 | 28.35 | 30.62 | 34.66 | 39.47 | 44.65 | 49.63 | 52.75 |
| 16.3 | 0.43 | 0.19 | 28.60 | 30.88 | 34.94 | 39.77 | 44.96 | 49.96 | 53.08 |
| 16.4 | 0.43 | 0.19 | 28.84 | 31.14 | 35.21 | 40.06 | 45.27 | 50.28 | 53.41 |
| 16.5 | 0.43 | 0.19 | 29.09 | 31.39 | 35.48 | 40.34 | 45.56 | 50.59 | 53.73 |
| 16.6 | 0.43 | 0.18 | 29.33 | 31.64 | 35.74 | 40.62 | 45.86 | 50.89 | 54.04 |
| 16.7 | 0.43 | 0.18 | 29.56 | 31.88 | 36.00 | 40.89 | 46.14 | 51.19 | 54.34 |
| 16.8 | 0.43 | 0.18 | 29.80 | 32.13 | 36.25 | 41.16 | 46.42 | 51.47 | 54.63 |
| 16.9 | 0.43 | 0.18 | 30.03 | 32.36 | 36.50 | 41.42 | 46.69 | 51.75 | 54.92 |
| 17.0 | 0.43 | 0.18 | 30.25 | 32.60 | 36.74 | 41.67 | 46.95 | 52.03 | 55.20 |
| 17.1 | 0.43 | 0.18 | 30.48 | 32.83 | 36.98 | 41.92 | 47.21 | 52.29 | 55.47 |
| 17.2 | 0.43 | 0.18 | 30.70 | 33.05 | 37.22 | 42.16 | 47.46 | 52.55 | 55.73 |
| 17.3 | 0.44 | 0.18 | 30.91 | 33.27 | 37.45 | 42.40 | 47.71 | 52.80 | 55.98 |
| 17.4 | 0.44 | 0.18 | 31.13 | 33.49 | 37.68 | 42.64 | 47.95 | 53.05 | 56.23 |
| 17.5 | 0.44 | 0.18 | 31.34 | 33.71 | 37.90 | 42.87 | 48.19 | 53.29 | 56.48 |
| 17.6 | 0.44 | 0.18 | 31.55 | 33.92 | 38.12 | 43.10 | 48.42 | 53.53 | 56.71 |
| 17.7 | 0.44 | 0.18 | 31.75 | 34.13 | 38.34 | 43.32 | 48.65 | 53.76 | 56.95 |
| 17.8 | 0.44 | 0.18 | 31.96 | 34.34 | 38.55 | 43.54 | 48.87 | 53.98 | 57.17 |
| 17.9 | 0.44 | 0.18 | 32.16 | 34.55 | 38.76 | 43.76 | 49.09 | 54.21 | 57.40 |
| 18.0 | 0.44 | 0.17 | 32.36 | 34.75 | 38.97 | 43.97 | 49.31 | 54.42 | 57.61 |
| 18.1 | 0.44 | 0.17 | 32.56 | 34.95 | 39.18 | 44.18 | 49.52 | 54.64 | 57.83 |
| 18.2 | 0.44 | 0.17 | 32.75 | 35.15 | 39.38 | 44.39 | 49.73 | 54.85 | 58.04 |
| 18.3 | 0.44 | 0.17 | 32.95 | 35.35 | 39.58 | 44.59 | 49.94 | 55.06 | 58.25 |
| 18.4 | 0.44 | 0.17 | 33.14 | 35.54 | 39.78 | 44.79 | 50.14 | 55.26 | 58.45 |
| 18.5 | 0.44 | 0.17 | 33.33 | 35.74 | 39.98 | 44.99 | 50.35 | 55.47 | 58.66 |
| 18.6 | 0.44 | 0.17 | 33.52 | 35.93 | 40.17 | 45.19 | 50.55 | 55.67 | 58.86 |
| 18.7 | 0.44 | 0.17 | 33.71 | 36.12 | 40.37 | 45.39 | 50.75 | 55.86 | 59.05 |
| 18.8 | 0.44 | 0.17 | 33.90 | 36.31 | 40.56 | 45.59 | 50.94 | 56.06 | 59.25 |
| 18.9 | 0.44 | 0.17 | 34.09 | 36.50 | 40.76 | 45.78 | 51.14 | 56.26 | 59.44 |
| 19.0 | 0.44 | 0.17 | 34.27 | 36.69 | 40.95 | 45.98 | 51.33 | 56.45 | 59.64 |
| 19.1 | 0.44 | 0.17 | 34.46 | 36.88 | 41.14 | 46.17 | 51.53 | 56.65 | 59.83 |
| 19.2 | 0.44 | 0.17 | 34.64 | 37.07 | 41.33 | 46.36 | 51.72 | 56.84 | 60.02 |
| 19.3 | 0.44 | 0.17 | 34.83 | 37.26 | 41.52 | 46.56 | 51.92 | 57.03 | 60.21 |
| 19.4 | 0.44 | 0.16 | 35.02 | 37.44 | 41.71 | 46.75 | 52.11 | 57.22 | 60.40 |
| 19.5 | 0.44 | 0.16 | 35.20 | 37.63 | 41.90 | 46.94 | 52.30 | 57.41 | 60.59 |
| 19.6 | 0.44 | 0.16 | 35.39 | 37.82 | 42.09 | 47.13 | 52.49 | 57.60 | 60.78 |
| 19.7 | 0.44 | 0.16 | 35.57 | 38.00 | 42.28 | 47.32 | 52.68 | 57.80 | 60.97 |
| 19.8 | 0.44 | 0.16 | 35.76 | 38.19 | 42.47 | 47.52 | 52.88 | 57.99 | 61.16 |
| 19.9 | 0.44 | 0.16 | 35.94 | 38.38 | 42.66 | 47.71 | 53.07 | 58.18 | 61.35 |

1. Non-dominant handgrip, kg

|  | Males | | | | | | | | |
| --- | --- | --- | --- | --- | --- | --- | --- | --- | --- |
| Age, y | L | S | 5th | 10th | 25th | 50th (M) | 75th | 90th | 95th |
| 6.0 | 0.23 | 0.23 | 6.53 | 7.15 | 8.29 | 9.70 | 11.30 | 12.91 | 13.95 |
| 6.1 | 0.24 | 0.23 | 6.63 | 7.26 | 8.42 | 9.85 | 11.47 | 13.10 | 14.16 |
| 6.2 | 0.24 | 0.23 | 6.74 | 7.37 | 8.55 | 10.01 | 11.65 | 13.30 | 14.37 |
| 6.3 | 0.24 | 0.23 | 6.84 | 7.49 | 8.68 | 10.16 | 11.82 | 13.49 | 14.58 |
| 6.4 | 0.24 | 0.23 | 6.94 | 7.60 | 8.81 | 10.31 | 11.99 | 13.69 | 14.79 |
| 6.5 | 0.24 | 0.23 | 7.05 | 7.71 | 8.94 | 10.46 | 12.17 | 13.88 | 14.99 |
| 6.6 | 0.24 | 0.23 | 7.15 | 7.83 | 9.07 | 10.61 | 12.34 | 14.08 | 15.20 |
| 6.7 | 0.24 | 0.23 | 7.26 | 7.94 | 9.20 | 10.76 | 12.51 | 14.27 | 15.41 |
| 6.8 | 0.24 | 0.23 | 7.36 | 8.06 | 9.33 | 10.91 | 12.69 | 14.47 | 15.62 |
| 6.9 | 0.24 | 0.23 | 7.47 | 8.17 | 9.46 | 11.06 | 12.86 | 14.66 | 15.83 |
| 7.0 | 0.24 | 0.23 | 7.57 | 8.28 | 9.59 | 11.21 | 13.03 | 14.86 | 16.04 |
| 7.1 | 0.25 | 0.23 | 7.68 | 8.40 | 9.72 | 11.36 | 13.20 | 15.05 | 16.25 |
| 7.2 | 0.25 | 0.23 | 7.78 | 8.51 | 9.85 | 11.51 | 13.38 | 15.25 | 16.45 |
| 7.3 | 0.25 | 0.23 | 7.89 | 8.63 | 9.98 | 11.67 | 13.55 | 15.44 | 16.66 |
| 7.4 | 0.25 | 0.23 | 8.00 | 8.74 | 10.12 | 11.82 | 13.73 | 15.64 | 16.88 |
| 7.5 | 0.25 | 0.23 | 8.10 | 8.86 | 10.25 | 11.97 | 13.90 | 15.84 | 17.09 |
| 7.6 | 0.25 | 0.23 | 8.21 | 8.98 | 10.38 | 12.13 | 14.08 | 16.04 | 17.30 |
| 7.7 | 0.25 | 0.23 | 8.32 | 9.10 | 10.52 | 12.28 | 14.26 | 16.24 | 17.51 |
| 7.8 | 0.26 | 0.23 | 8.43 | 9.22 | 10.66 | 12.44 | 14.44 | 16.44 | 17.73 |
| 7.9 | 0.26 | 0.22 | 8.54 | 9.34 | 10.79 | 12.60 | 14.62 | 16.64 | 17.94 |
| 8.0 | 0.26 | 0.22 | 8.65 | 9.46 | 10.93 | 12.76 | 14.80 | 16.85 | 18.16 |
| 8.1 | 0.26 | 0.22 | 8.76 | 9.58 | 11.07 | 12.92 | 14.99 | 17.05 | 18.38 |
| 8.2 | 0.26 | 0.22 | 8.88 | 9.71 | 11.22 | 13.09 | 15.17 | 17.26 | 18.60 |
| 8.3 | 0.27 | 0.22 | 9.00 | 9.83 | 11.36 | 13.25 | 15.36 | 17.47 | 18.83 |
| 8.4 | 0.27 | 0.22 | 9.11 | 9.96 | 11.51 | 13.42 | 15.56 | 17.69 | 19.06 |
| 8.5 | 0.27 | 0.22 | 9.24 | 10.09 | 11.66 | 13.59 | 15.75 | 17.91 | 19.29 |
| 8.6 | 0.27 | 0.22 | 9.36 | 10.23 | 11.81 | 13.77 | 15.96 | 18.13 | 19.53 |
| 8.7 | 0.27 | 0.22 | 9.48 | 10.36 | 11.97 | 13.95 | 16.16 | 18.36 | 19.77 |
| 8.8 | 0.28 | 0.22 | 9.61 | 10.50 | 12.13 | 14.13 | 16.37 | 18.59 | 20.02 |
| 8.9 | 0.28 | 0.22 | 9.74 | 10.64 | 12.29 | 14.32 | 16.58 | 18.83 | 20.27 |
| 9.0 | 0.28 | 0.22 | 9.87 | 10.79 | 12.45 | 14.51 | 16.79 | 19.07 | 20.53 |
| 9.1 | 0.28 | 0.22 | 10.01 | 10.93 | 12.62 | 14.70 | 17.01 | 19.31 | 20.79 |
| 9.2 | 0.29 | 0.22 | 10.15 | 11.08 | 12.79 | 14.90 | 17.24 | 19.56 | 21.05 |
| 9.3 | 0.29 | 0.22 | 10.29 | 11.24 | 12.97 | 15.10 | 17.46 | 19.81 | 21.32 |
| 9.4 | 0.29 | 0.22 | 10.43 | 11.39 | 13.14 | 15.30 | 17.70 | 20.07 | 21.60 |
| 9.5 | 0.29 | 0.22 | 10.57 | 11.55 | 13.32 | 15.51 | 17.93 | 20.34 | 21.88 |
| 9.6 | 0.30 | 0.22 | 10.72 | 11.71 | 13.51 | 15.72 | 18.17 | 20.61 | 22.16 |
| 9.7 | 0.30 | 0.22 | 10.87 | 11.88 | 13.70 | 15.94 | 18.42 | 20.88 | 22.46 |
| 9.8 | 0.30 | 0.22 | 11.03 | 12.04 | 13.89 | 16.16 | 18.67 | 21.16 | 22.75 |
| 9.9 | 0.30 | 0.22 | 11.19 | 12.22 | 14.08 | 16.38 | 18.92 | 21.44 | 23.06 |
| 10.0 | 0.31 | 0.22 | 11.35 | 12.39 | 14.28 | 16.61 | 19.19 | 21.74 | 23.36 |
| 10.1 | 0.31 | 0.22 | 11.51 | 12.57 | 14.49 | 16.84 | 19.45 | 22.03 | 23.68 |
| 10.2 | 0.31 | 0.22 | 11.68 | 12.75 | 14.69 | 17.08 | 19.72 | 22.33 | 24.00 |
| 10.3 | 0.31 | 0.22 | 11.85 | 12.93 | 14.91 | 17.32 | 20.00 | 22.64 | 24.33 |
| 10.4 | 0.32 | 0.22 | 12.02 | 13.12 | 15.12 | 17.57 | 20.28 | 22.96 | 24.66 |
| 10.5 | 0.32 | 0.22 | 12.20 | 13.31 | 15.34 | 17.83 | 20.57 | 23.28 | 25.01 |
| 10.6 | 0.32 | 0.22 | 12.38 | 13.51 | 15.57 | 18.08 | 20.86 | 23.61 | 25.36 |
| 10.7 | 0.32 | 0.22 | 12.56 | 13.71 | 15.80 | 18.35 | 21.16 | 23.94 | 25.71 |
| 10.8 | 0.33 | 0.22 | 12.75 | 13.92 | 16.03 | 18.62 | 21.47 | 24.28 | 26.08 |
| 10.9 | 0.33 | 0.22 | 12.94 | 14.13 | 16.27 | 18.89 | 21.78 | 24.63 | 26.45 |
| 11.0 | 0.33 | 0.22 | 13.14 | 14.34 | 16.52 | 19.18 | 22.10 | 24.99 | 26.83 |
| 11.1 | 0.33 | 0.22 | 13.34 | 14.56 | 16.77 | 19.46 | 22.43 | 25.36 | 27.22 |
| 11.2 | 0.34 | 0.22 | 13.54 | 14.78 | 17.02 | 19.76 | 22.76 | 25.73 | 27.61 |
| 11.3 | 0.34 | 0.22 | 13.75 | 15.01 | 17.28 | 20.05 | 23.10 | 26.11 | 28.01 |
| 11.4 | 0.34 | 0.21 | 13.96 | 15.24 | 17.55 | 20.36 | 23.45 | 26.49 | 28.42 |
| 11.5 | 0.35 | 0.21 | 14.18 | 15.48 | 17.82 | 20.67 | 23.80 | 26.88 | 28.84 |
| 11.6 | 0.35 | 0.21 | 14.40 | 15.71 | 18.09 | 20.98 | 24.16 | 27.28 | 29.26 |
| 11.7 | 0.35 | 0.21 | 14.62 | 15.96 | 18.37 | 21.30 | 24.52 | 27.69 | 29.69 |
| 11.8 | 0.35 | 0.21 | 14.85 | 16.21 | 18.65 | 21.63 | 24.89 | 28.10 | 30.13 |
| 11.9 | 0.36 | 0.21 | 15.08 | 16.46 | 18.94 | 21.96 | 25.27 | 28.51 | 30.57 |
| 12.0 | 0.36 | 0.21 | 15.32 | 16.71 | 19.23 | 22.29 | 25.65 | 28.94 | 31.02 |
| 12.1 | 0.36 | 0.21 | 15.55 | 16.97 | 19.53 | 22.63 | 26.03 | 29.37 | 31.48 |
| 12.2 | 0.36 | 0.21 | 15.80 | 17.24 | 19.83 | 22.98 | 26.42 | 29.80 | 31.94 |
| 12.3 | 0.37 | 0.21 | 16.04 | 17.50 | 20.13 | 23.33 | 26.82 | 30.24 | 32.41 |
| 12.4 | 0.37 | 0.21 | 16.29 | 17.77 | 20.44 | 23.68 | 27.22 | 30.69 | 32.89 |
| 12.5 | 0.37 | 0.21 | 16.55 | 18.05 | 20.76 | 24.04 | 27.63 | 31.14 | 33.37 |
| 12.6 | 0.38 | 0.21 | 16.80 | 18.33 | 21.07 | 24.40 | 28.04 | 31.60 | 33.85 |
| 12.7 | 0.38 | 0.21 | 17.06 | 18.61 | 21.40 | 24.77 | 28.46 | 32.06 | 34.34 |
| 12.8 | 0.38 | 0.21 | 17.32 | 18.90 | 21.72 | 25.14 | 28.88 | 32.53 | 34.84 |
| 12.9 | 0.38 | 0.21 | 17.59 | 19.18 | 22.05 | 25.52 | 29.30 | 33.00 | 35.33 |
| 13.0 | 0.39 | 0.21 | 17.86 | 19.48 | 22.38 | 25.89 | 29.73 | 33.47 | 35.83 |
| 13.1 | 0.39 | 0.21 | 18.13 | 19.77 | 22.71 | 26.27 | 30.16 | 33.94 | 36.34 |
| 13.2 | 0.39 | 0.21 | 18.40 | 20.06 | 23.05 | 26.65 | 30.59 | 34.42 | 36.84 |
| 13.3 | 0.39 | 0.21 | 18.68 | 20.36 | 23.38 | 27.04 | 31.02 | 34.89 | 37.34 |
| 13.4 | 0.40 | 0.21 | 18.95 | 20.66 | 23.72 | 27.42 | 31.45 | 35.37 | 37.85 |
| 13.5 | 0.40 | 0.21 | 19.23 | 20.96 | 24.06 | 27.80 | 31.88 | 35.85 | 38.35 |
| 13.6 | 0.40 | 0.21 | 19.50 | 21.26 | 24.40 | 28.19 | 32.31 | 36.32 | 38.85 |
| 13.7 | 0.40 | 0.21 | 19.78 | 21.56 | 24.74 | 28.57 | 32.74 | 36.80 | 39.35 |
| 13.8 | 0.41 | 0.21 | 20.06 | 21.86 | 25.08 | 28.96 | 33.17 | 37.27 | 39.85 |
| 13.9 | 0.41 | 0.21 | 20.34 | 22.16 | 25.42 | 29.34 | 33.60 | 37.74 | 40.34 |
| 14.0 | 0.41 | 0.21 | 20.62 | 22.46 | 25.75 | 29.72 | 34.02 | 38.20 | 40.84 |
| 14.1 | 0.41 | 0.21 | 20.90 | 22.76 | 26.09 | 30.10 | 34.45 | 38.67 | 41.33 |
| 14.2 | 0.42 | 0.21 | 21.17 | 23.06 | 26.43 | 30.48 | 34.87 | 39.13 | 41.81 |
| 14.3 | 0.42 | 0.20 | 21.45 | 23.36 | 26.76 | 30.85 | 35.29 | 39.58 | 42.29 |
| 14.4 | 0.42 | 0.20 | 21.73 | 23.65 | 27.09 | 31.23 | 35.70 | 40.04 | 42.76 |
| 14.5 | 0.43 | 0.20 | 22.00 | 23.95 | 27.42 | 31.60 | 36.11 | 40.48 | 43.23 |
| 14.6 | 0.43 | 0.20 | 22.27 | 24.24 | 27.75 | 31.96 | 36.52 | 40.92 | 43.69 |
| 14.7 | 0.43 | 0.20 | 22.55 | 24.53 | 28.08 | 32.32 | 36.92 | 41.36 | 44.15 |
| 14.8 | 0.43 | 0.20 | 22.82 | 24.82 | 28.40 | 32.68 | 37.31 | 41.79 | 44.60 |
| 14.9 | 0.44 | 0.20 | 23.08 | 25.11 | 28.72 | 33.04 | 37.70 | 42.21 | 45.04 |
| 15.0 | 0.44 | 0.20 | 23.35 | 25.39 | 29.03 | 33.39 | 38.09 | 42.63 | 45.47 |
| 15.1 | 0.44 | 0.20 | 23.61 | 25.67 | 29.34 | 33.73 | 38.47 | 43.04 | 45.90 |
| 15.2 | 0.45 | 0.20 | 23.87 | 25.95 | 29.65 | 34.07 | 38.84 | 43.44 | 46.32 |
| 15.3 | 0.45 | 0.20 | 24.13 | 26.22 | 29.95 | 34.41 | 39.21 | 43.83 | 46.73 |
| 15.4 | 0.45 | 0.20 | 24.38 | 26.49 | 30.25 | 34.74 | 39.57 | 44.22 | 47.13 |
| 15.5 | 0.45 | 0.20 | 24.63 | 26.76 | 30.55 | 35.06 | 39.92 | 44.60 | 47.52 |
| 15.6 | 0.46 | 0.20 | 24.88 | 27.03 | 30.84 | 35.38 | 40.27 | 44.97 | 47.91 |
| 15.7 | 0.46 | 0.20 | 25.12 | 27.29 | 31.12 | 35.69 | 40.61 | 45.33 | 48.29 |
| 15.8 | 0.46 | 0.20 | 25.37 | 27.54 | 31.40 | 36.00 | 40.94 | 45.69 | 48.65 |
| 15.9 | 0.47 | 0.20 | 25.60 | 27.79 | 31.68 | 36.30 | 41.27 | 46.03 | 49.01 |
| 16.0 | 0.47 | 0.20 | 25.84 | 28.04 | 31.95 | 36.60 | 41.59 | 46.37 | 49.36 |
| 16.1 | 0.47 | 0.19 | 26.07 | 28.29 | 32.22 | 36.89 | 41.90 | 46.70 | 49.70 |
| 16.2 | 0.48 | 0.19 | 26.30 | 28.53 | 32.48 | 37.18 | 42.21 | 47.03 | 50.04 |
| 16.3 | 0.48 | 0.19 | 26.52 | 28.77 | 32.74 | 37.46 | 42.51 | 47.34 | 50.36 |
| 16.4 | 0.49 | 0.19 | 26.74 | 29.00 | 33.00 | 37.73 | 42.80 | 47.65 | 50.68 |
| 16.5 | 0.49 | 0.19 | 26.96 | 29.23 | 33.24 | 38.00 | 43.09 | 47.95 | 50.99 |
| 16.6 | 0.49 | 0.19 | 27.17 | 29.46 | 33.49 | 38.27 | 43.37 | 48.25 | 51.29 |
| 16.7 | 0.50 | 0.19 | 27.38 | 29.68 | 33.73 | 38.53 | 43.64 | 48.54 | 51.58 |
| 16.8 | 0.50 | 0.19 | 27.59 | 29.90 | 33.97 | 38.78 | 43.91 | 48.82 | 51.87 |
| 16.9 | 0.51 | 0.19 | 27.80 | 30.11 | 34.20 | 39.03 | 44.18 | 49.09 | 52.15 |
| 17.0 | 0.51 | 0.19 | 28.00 | 30.33 | 34.43 | 39.28 | 44.44 | 49.36 | 52.42 |
| 17.1 | 0.51 | 0.19 | 28.20 | 30.54 | 34.66 | 39.52 | 44.69 | 49.62 | 52.69 |
| 17.2 | 0.52 | 0.19 | 28.39 | 30.74 | 34.88 | 39.76 | 44.94 | 49.88 | 52.95 |
| 17.3 | 0.52 | 0.19 | 28.59 | 30.95 | 35.10 | 39.99 | 45.19 | 50.13 | 53.20 |
| 17.4 | 0.53 | 0.19 | 28.78 | 31.15 | 35.31 | 40.22 | 45.43 | 50.38 | 53.45 |
| 17.5 | 0.53 | 0.19 | 28.97 | 31.35 | 35.53 | 40.45 | 45.66 | 50.62 | 53.69 |
| 17.6 | 0.54 | 0.19 | 29.15 | 31.54 | 35.74 | 40.67 | 45.89 | 50.86 | 53.93 |
| 17.7 | 0.54 | 0.18 | 29.34 | 31.73 | 35.94 | 40.89 | 46.12 | 51.09 | 54.17 |
| 17.8 | 0.55 | 0.18 | 29.52 | 31.93 | 36.15 | 41.11 | 46.35 | 51.32 | 54.40 |
| 17.9 | 0.55 | 0.18 | 29.70 | 32.11 | 36.35 | 41.32 | 46.57 | 51.55 | 54.63 |
| 18.0 | 0.56 | 0.18 | 29.88 | 32.30 | 36.55 | 41.53 | 46.79 | 51.77 | 54.85 |
| 18.1 | 0.56 | 0.18 | 30.05 | 32.49 | 36.75 | 41.74 | 47.01 | 51.99 | 55.07 |
| 18.2 | 0.57 | 0.18 | 30.23 | 32.67 | 36.95 | 41.95 | 47.23 | 52.21 | 55.29 |
| 18.3 | 0.58 | 0.18 | 30.40 | 32.85 | 37.14 | 42.16 | 47.44 | 52.43 | 55.51 |
| 18.4 | 0.58 | 0.18 | 30.57 | 33.03 | 37.34 | 42.36 | 47.65 | 52.64 | 55.72 |
| 18.5 | 0.59 | 0.18 | 30.74 | 33.21 | 37.53 | 42.57 | 47.86 | 52.85 | 55.93 |
| 18.6 | 0.59 | 0.18 | 30.91 | 33.39 | 37.72 | 42.77 | 48.07 | 53.06 | 56.14 |
| 18.7 | 0.60 | 0.18 | 31.08 | 33.57 | 37.91 | 42.97 | 48.28 | 53.27 | 56.35 |
| 18.8 | 0.61 | 0.18 | 31.25 | 33.75 | 38.10 | 43.17 | 48.48 | 53.48 | 56.56 |
| 18.9 | 0.61 | 0.18 | 31.41 | 33.92 | 38.29 | 43.37 | 48.69 | 53.69 | 56.76 |
| 19.0 | 0.62 | 0.18 | 31.58 | 34.10 | 38.48 | 43.56 | 48.89 | 53.89 | 56.97 |
| 19.1 | 0.62 | 0.18 | 31.75 | 34.27 | 38.66 | 43.76 | 49.09 | 54.10 | 57.17 |
| 19.2 | 0.63 | 0.18 | 31.91 | 34.45 | 38.85 | 43.96 | 49.29 | 54.30 | 57.37 |
| 19.3 | 0.64 | 0.18 | 32.08 | 34.62 | 39.04 | 44.15 | 49.50 | 54.50 | 57.58 |
| 19.4 | 0.64 | 0.18 | 32.24 | 34.80 | 39.22 | 44.35 | 49.70 | 54.71 | 57.78 |
| 19.5 | 0.65 | 0.17 | 32.41 | 34.97 | 39.41 | 44.55 | 49.90 | 54.91 | 57.98 |
| 19.6 | 0.65 | 0.17 | 32.58 | 35.15 | 39.60 | 44.74 | 50.10 | 55.11 | 58.18 |
| 19.7 | 0.66 | 0.17 | 32.74 | 35.32 | 39.78 | 44.94 | 50.30 | 55.31 | 58.38 |
| 19.8 | 0.67 | 0.17 | 32.91 | 35.50 | 39.97 | 45.13 | 50.50 | 55.52 | 58.58 |
| 19.9 | 0.67 | 0.17 | 33.07 | 35.67 | 40.16 | 45.33 | 50.70 | 55.72 | 58.78 |

1. Upper arm length, cm

|  | Males | | | | | | | | |
| --- | --- | --- | --- | --- | --- | --- | --- | --- | --- |
| Age, y | L | S | 5th | 10th | 25th | 50th (M) | 75th | 90th | 95th |
| 6.0 | 0.20 | 0.07 | 21.68 | 22.21 | 23.12 | 24.17 | 25.25 | 26.26 | 26.88 |
| 6.1 | 0.21 | 0.07 | 21.81 | 22.35 | 23.26 | 24.32 | 25.40 | 26.42 | 27.04 |
| 6.2 | 0.21 | 0.07 | 21.94 | 22.48 | 23.40 | 24.46 | 25.56 | 26.58 | 27.21 |
| 6.3 | 0.22 | 0.07 | 22.07 | 22.61 | 23.54 | 24.61 | 25.71 | 26.74 | 27.37 |
| 6.4 | 0.22 | 0.07 | 22.20 | 22.74 | 23.68 | 24.75 | 25.87 | 26.90 | 27.54 |
| 6.5 | 0.23 | 0.07 | 22.32 | 22.87 | 23.82 | 24.90 | 26.02 | 27.06 | 27.70 |
| 6.6 | 0.23 | 0.07 | 22.45 | 23.01 | 23.96 | 25.05 | 26.17 | 27.22 | 27.87 |
| 6.7 | 0.24 | 0.07 | 22.58 | 23.14 | 24.10 | 25.19 | 26.33 | 27.39 | 28.03 |
| 6.8 | 0.24 | 0.07 | 22.71 | 23.27 | 24.23 | 25.34 | 26.48 | 27.55 | 28.20 |
| 6.9 | 0.25 | 0.07 | 22.83 | 23.40 | 24.37 | 25.49 | 26.64 | 27.71 | 28.36 |
| 7.0 | 0.25 | 0.07 | 22.96 | 23.53 | 24.51 | 25.63 | 26.79 | 27.87 | 28.53 |
| 7.1 | 0.25 | 0.07 | 23.09 | 23.66 | 24.65 | 25.78 | 26.94 | 28.03 | 28.69 |
| 7.2 | 0.26 | 0.07 | 23.22 | 23.80 | 24.79 | 25.92 | 27.10 | 28.19 | 28.86 |
| 7.3 | 0.26 | 0.07 | 23.34 | 23.93 | 24.93 | 26.07 | 27.25 | 28.35 | 29.02 |
| 7.4 | 0.27 | 0.07 | 23.47 | 24.06 | 25.06 | 26.21 | 27.40 | 28.51 | 29.19 |
| 7.5 | 0.27 | 0.07 | 23.60 | 24.19 | 25.20 | 26.36 | 27.56 | 28.67 | 29.35 |
| 7.6 | 0.27 | 0.07 | 23.73 | 24.32 | 25.34 | 26.51 | 27.71 | 28.83 | 29.52 |
| 7.7 | 0.28 | 0.07 | 23.85 | 24.45 | 25.48 | 26.65 | 27.86 | 28.99 | 29.68 |
| 7.8 | 0.28 | 0.07 | 23.98 | 24.58 | 25.62 | 26.80 | 28.02 | 29.15 | 29.85 |
| 7.9 | 0.28 | 0.07 | 24.11 | 24.72 | 25.76 | 26.94 | 28.17 | 29.31 | 30.01 |
| 8.0 | 0.28 | 0.07 | 24.24 | 24.85 | 25.89 | 27.09 | 28.33 | 29.47 | 30.18 |
| 8.1 | 0.28 | 0.07 | 24.37 | 24.98 | 26.03 | 27.24 | 28.48 | 29.64 | 30.34 |
| 8.2 | 0.29 | 0.07 | 24.49 | 25.11 | 26.17 | 27.38 | 28.63 | 29.80 | 30.51 |
| 8.3 | 0.29 | 0.07 | 24.62 | 25.24 | 26.31 | 27.53 | 28.79 | 29.96 | 30.67 |
| 8.4 | 0.29 | 0.07 | 24.75 | 25.38 | 26.45 | 27.68 | 28.94 | 30.12 | 30.84 |
| 8.5 | 0.29 | 0.07 | 24.88 | 25.51 | 26.59 | 27.82 | 29.10 | 30.28 | 31.00 |
| 8.6 | 0.29 | 0.07 | 25.01 | 25.64 | 26.73 | 27.97 | 29.25 | 30.44 | 31.17 |
| 8.7 | 0.29 | 0.07 | 25.14 | 25.78 | 26.87 | 28.12 | 29.41 | 30.61 | 31.34 |
| 8.8 | 0.28 | 0.07 | 25.27 | 25.91 | 27.01 | 28.26 | 29.56 | 30.77 | 31.50 |
| 8.9 | 0.28 | 0.07 | 25.40 | 26.04 | 27.15 | 28.41 | 29.72 | 30.93 | 31.67 |
| 9.0 | 0.28 | 0.07 | 25.53 | 26.18 | 27.29 | 28.56 | 29.87 | 31.10 | 31.84 |
| 9.1 | 0.27 | 0.07 | 25.66 | 26.31 | 27.43 | 28.71 | 30.03 | 31.26 | 32.01 |
| 9.2 | 0.27 | 0.07 | 25.80 | 26.45 | 27.57 | 28.86 | 30.19 | 31.42 | 32.18 |
| 9.3 | 0.26 | 0.07 | 25.93 | 26.59 | 27.72 | 29.01 | 30.35 | 31.59 | 32.35 |
| 9.4 | 0.26 | 0.07 | 26.06 | 26.72 | 27.86 | 29.16 | 30.50 | 31.76 | 32.52 |
| 9.5 | 0.25 | 0.07 | 26.20 | 26.86 | 28.00 | 29.31 | 30.66 | 31.92 | 32.69 |
| 9.6 | 0.25 | 0.07 | 26.33 | 27.00 | 28.15 | 29.46 | 30.82 | 32.09 | 32.86 |
| 9.7 | 0.24 | 0.07 | 26.47 | 27.14 | 28.29 | 29.61 | 30.98 | 32.26 | 33.04 |
| 9.8 | 0.23 | 0.07 | 26.60 | 27.28 | 28.44 | 29.76 | 31.14 | 32.42 | 33.21 |
| 9.9 | 0.22 | 0.07 | 26.74 | 27.42 | 28.58 | 29.92 | 31.30 | 32.59 | 33.38 |
| 10.0 | 0.21 | 0.07 | 26.87 | 27.56 | 28.73 | 30.07 | 31.46 | 32.76 | 33.55 |
| 10.1 | 0.20 | 0.07 | 27.01 | 27.70 | 28.87 | 30.22 | 31.62 | 32.93 | 33.73 |
| 10.2 | 0.19 | 0.07 | 27.15 | 27.84 | 29.02 | 30.37 | 31.78 | 33.10 | 33.90 |
| 10.3 | 0.17 | 0.07 | 27.29 | 27.98 | 29.16 | 30.53 | 31.94 | 33.27 | 34.08 |
| 10.4 | 0.16 | 0.07 | 27.43 | 28.12 | 29.31 | 30.68 | 32.10 | 33.43 | 34.25 |
| 10.5 | 0.15 | 0.07 | 27.56 | 28.26 | 29.46 | 30.83 | 32.26 | 33.60 | 34.43 |
| 10.6 | 0.14 | 0.07 | 27.70 | 28.40 | 29.60 | 30.99 | 32.43 | 33.77 | 34.60 |
| 10.7 | 0.12 | 0.07 | 27.84 | 28.54 | 29.75 | 31.14 | 32.59 | 33.94 | 34.77 |
| 10.8 | 0.11 | 0.07 | 27.98 | 28.68 | 29.90 | 31.29 | 32.75 | 34.11 | 34.95 |
| 10.9 | 0.09 | 0.07 | 28.12 | 28.83 | 30.04 | 31.45 | 32.91 | 34.28 | 35.12 |
| 11.0 | 0.08 | 0.07 | 28.26 | 28.97 | 30.19 | 31.60 | 33.07 | 34.45 | 35.30 |
| 11.1 | 0.06 | 0.07 | 28.40 | 29.11 | 30.34 | 31.75 | 33.23 | 34.62 | 35.47 |
| 11.2 | 0.05 | 0.07 | 28.54 | 29.25 | 30.48 | 31.90 | 33.39 | 34.78 | 35.64 |
| 11.3 | 0.03 | 0.07 | 28.68 | 29.39 | 30.63 | 32.05 | 33.55 | 34.95 | 35.81 |
| 11.4 | 0.02 | 0.07 | 28.82 | 29.53 | 30.77 | 32.21 | 33.70 | 35.12 | 35.99 |
| 11.5 | 0.00 | 0.07 | 28.96 | 29.68 | 30.92 | 32.36 | 33.86 | 35.28 | 36.16 |
| 11.6 | -0.01 | 0.07 | 29.10 | 29.82 | 31.06 | 32.51 | 34.02 | 35.44 | 36.32 |
| 11.7 | -0.03 | 0.07 | 29.23 | 29.96 | 31.21 | 32.66 | 34.18 | 35.61 | 36.49 |
| 11.8 | -0.04 | 0.07 | 29.37 | 30.09 | 31.35 | 32.80 | 34.33 | 35.77 | 36.66 |
| 11.9 | -0.06 | 0.07 | 29.51 | 30.23 | 31.49 | 32.95 | 34.48 | 35.93 | 36.83 |
| 12.0 | -0.07 | 0.07 | 29.64 | 30.37 | 31.63 | 33.10 | 34.64 | 36.09 | 36.99 |
| 12.1 | -0.09 | 0.07 | 29.78 | 30.51 | 31.77 | 33.24 | 34.79 | 36.25 | 37.15 |
| 12.2 | -0.10 | 0.07 | 29.91 | 30.64 | 31.91 | 33.39 | 34.94 | 36.41 | 37.32 |
| 12.3 | -0.12 | 0.07 | 30.05 | 30.78 | 32.05 | 33.53 | 35.09 | 36.56 | 37.48 |
| 12.4 | -0.13 | 0.07 | 30.18 | 30.91 | 32.19 | 33.67 | 35.24 | 36.72 | 37.63 |
| 12.5 | -0.15 | 0.07 | 30.31 | 31.05 | 32.33 | 33.81 | 35.38 | 36.87 | 37.79 |
| 12.6 | -0.16 | 0.07 | 30.44 | 31.18 | 32.46 | 33.95 | 35.53 | 37.02 | 37.95 |
| 12.7 | -0.18 | 0.07 | 30.57 | 31.31 | 32.59 | 34.09 | 35.67 | 37.17 | 38.10 |
| 12.8 | -0.19 | 0.07 | 30.70 | 31.44 | 32.73 | 34.23 | 35.81 | 37.32 | 38.25 |
| 12.9 | -0.21 | 0.07 | 30.83 | 31.57 | 32.86 | 34.36 | 35.95 | 37.46 | 38.40 |
| 13.0 | -0.22 | 0.07 | 30.95 | 31.69 | 32.99 | 34.49 | 36.09 | 37.60 | 38.54 |
| 13.1 | -0.23 | 0.07 | 31.07 | 31.82 | 33.11 | 34.62 | 36.22 | 37.74 | 38.69 |
| 13.2 | -0.25 | 0.07 | 31.20 | 31.94 | 33.24 | 34.75 | 36.36 | 37.88 | 38.83 |
| 13.3 | -0.26 | 0.07 | 31.32 | 32.06 | 33.36 | 34.88 | 36.49 | 38.02 | 38.97 |
| 13.4 | -0.27 | 0.07 | 31.44 | 32.18 | 33.48 | 35.01 | 36.62 | 38.15 | 39.11 |
| 13.5 | -0.28 | 0.07 | 31.55 | 32.30 | 33.60 | 35.13 | 36.74 | 38.28 | 39.24 |
| 13.6 | -0.29 | 0.07 | 31.67 | 32.42 | 33.72 | 35.25 | 36.87 | 38.41 | 39.37 |
| 13.7 | -0.31 | 0.07 | 31.78 | 32.53 | 33.84 | 35.37 | 36.99 | 38.53 | 39.50 |
| 13.8 | -0.32 | 0.07 | 31.89 | 32.64 | 33.95 | 35.48 | 37.11 | 38.66 | 39.62 |
| 13.9 | -0.33 | 0.07 | 32.00 | 32.75 | 34.06 | 35.60 | 37.22 | 38.78 | 39.75 |
| 14.0 | -0.34 | 0.07 | 32.11 | 32.86 | 34.17 | 35.71 | 37.34 | 38.89 | 39.87 |
| 14.1 | -0.35 | 0.07 | 32.21 | 32.96 | 34.28 | 35.82 | 37.45 | 39.01 | 39.98 |
| 14.2 | -0.35 | 0.07 | 32.32 | 33.07 | 34.38 | 35.92 | 37.56 | 39.12 | 40.10 |
| 14.3 | -0.36 | 0.07 | 32.42 | 33.17 | 34.48 | 36.03 | 37.66 | 39.23 | 40.21 |
| 14.4 | -0.37 | 0.07 | 32.52 | 33.27 | 34.58 | 36.13 | 37.77 | 39.34 | 40.32 |
| 14.5 | -0.38 | 0.07 | 32.61 | 33.37 | 34.68 | 36.23 | 37.87 | 39.44 | 40.42 |
| 14.6 | -0.38 | 0.07 | 32.71 | 33.46 | 34.78 | 36.32 | 37.97 | 39.54 | 40.52 |
| 14.7 | -0.39 | 0.06 | 32.80 | 33.55 | 34.87 | 36.42 | 38.06 | 39.64 | 40.62 |
| 14.8 | -0.39 | 0.06 | 32.89 | 33.64 | 34.96 | 36.51 | 38.16 | 39.73 | 40.72 |
| 14.9 | -0.40 | 0.06 | 32.97 | 33.73 | 35.05 | 36.60 | 38.25 | 39.82 | 40.81 |
| 15.0 | -0.40 | 0.06 | 33.06 | 33.81 | 35.14 | 36.69 | 38.33 | 39.91 | 40.90 |
| 15.1 | -0.40 | 0.06 | 33.14 | 33.90 | 35.22 | 36.77 | 38.42 | 40.00 | 40.98 |
| 15.2 | -0.40 | 0.06 | 33.22 | 33.98 | 35.30 | 36.85 | 38.50 | 40.08 | 41.07 |
| 15.3 | -0.40 | 0.06 | 33.30 | 34.06 | 35.38 | 36.93 | 38.58 | 40.16 | 41.15 |
| 15.4 | -0.40 | 0.06 | 33.37 | 34.13 | 35.46 | 37.01 | 38.66 | 40.24 | 41.22 |
| 15.5 | -0.40 | 0.06 | 33.45 | 34.21 | 35.53 | 37.08 | 38.73 | 40.31 | 41.30 |
| 15.6 | -0.40 | 0.06 | 33.52 | 34.28 | 35.60 | 37.16 | 38.81 | 40.38 | 41.37 |
| 15.7 | -0.40 | 0.06 | 33.59 | 34.35 | 35.67 | 37.23 | 38.88 | 40.45 | 41.44 |
| 15.8 | -0.40 | 0.06 | 33.65 | 34.41 | 35.74 | 37.29 | 38.94 | 40.52 | 41.51 |
| 15.9 | -0.39 | 0.06 | 33.72 | 34.48 | 35.80 | 37.36 | 39.01 | 40.58 | 41.57 |
| 16.0 | -0.39 | 0.06 | 33.78 | 34.54 | 35.87 | 37.42 | 39.07 | 40.65 | 41.63 |
| 16.1 | -0.38 | 0.06 | 33.84 | 34.60 | 35.93 | 37.48 | 39.13 | 40.71 | 41.69 |
| 16.2 | -0.37 | 0.06 | 33.90 | 34.66 | 35.99 | 37.54 | 39.19 | 40.76 | 41.74 |
| 16.3 | -0.37 | 0.06 | 33.95 | 34.71 | 36.04 | 37.60 | 39.24 | 40.82 | 41.80 |
| 16.4 | -0.36 | 0.06 | 34.01 | 34.77 | 36.10 | 37.65 | 39.30 | 40.87 | 41.85 |
| 16.5 | -0.35 | 0.06 | 34.06 | 34.82 | 36.15 | 37.70 | 39.35 | 40.92 | 41.89 |
| 16.6 | -0.34 | 0.06 | 34.11 | 34.87 | 36.20 | 37.75 | 39.40 | 40.96 | 41.94 |
| 16.7 | -0.33 | 0.06 | 34.16 | 34.92 | 36.25 | 37.80 | 39.45 | 41.01 | 41.98 |
| 16.8 | -0.32 | 0.06 | 34.20 | 34.97 | 36.30 | 37.85 | 39.49 | 41.05 | 42.03 |
| 16.9 | -0.31 | 0.06 | 34.25 | 35.01 | 36.34 | 37.89 | 39.53 | 41.09 | 42.07 |
| 17.0 | -0.30 | 0.06 | 34.29 | 35.05 | 36.38 | 37.94 | 39.58 | 41.13 | 42.10 |
| 17.1 | -0.28 | 0.06 | 34.33 | 35.10 | 36.43 | 37.98 | 39.62 | 41.17 | 42.14 |
| 17.2 | -0.27 | 0.06 | 34.37 | 35.14 | 36.47 | 38.02 | 39.65 | 41.21 | 42.17 |
| 17.3 | -0.26 | 0.06 | 34.41 | 35.17 | 36.51 | 38.06 | 39.69 | 41.24 | 42.21 |
| 17.4 | -0.24 | 0.06 | 34.45 | 35.21 | 36.54 | 38.09 | 39.73 | 41.28 | 42.24 |
| 17.5 | -0.23 | 0.06 | 34.48 | 35.25 | 36.58 | 38.13 | 39.76 | 41.31 | 42.27 |
| 17.6 | -0.21 | 0.06 | 34.52 | 35.28 | 36.62 | 38.16 | 39.79 | 41.34 | 42.29 |
| 17.7 | -0.20 | 0.06 | 34.55 | 35.32 | 36.65 | 38.20 | 39.83 | 41.37 | 42.32 |
| 17.8 | -0.18 | 0.06 | 34.58 | 35.35 | 36.68 | 38.23 | 39.86 | 41.39 | 42.35 |
| 17.9 | -0.16 | 0.06 | 34.61 | 35.38 | 36.72 | 38.26 | 39.89 | 41.42 | 42.37 |
| 18.0 | -0.15 | 0.06 | 34.64 | 35.41 | 36.75 | 38.29 | 39.92 | 41.45 | 42.39 |
| 18.1 | -0.13 | 0.06 | 34.67 | 35.44 | 36.78 | 38.32 | 39.94 | 41.47 | 42.42 |
| 18.2 | -0.11 | 0.06 | 34.70 | 35.47 | 36.81 | 38.35 | 39.97 | 41.50 | 42.44 |
| 18.3 | -0.10 | 0.06 | 34.73 | 35.50 | 36.84 | 38.38 | 40.00 | 41.52 | 42.46 |
| 18.4 | -0.08 | 0.06 | 34.76 | 35.53 | 36.86 | 38.41 | 40.03 | 41.55 | 42.48 |
| 18.5 | -0.06 | 0.06 | 34.78 | 35.56 | 36.89 | 38.44 | 40.05 | 41.57 | 42.50 |
| 18.6 | -0.04 | 0.06 | 34.81 | 35.58 | 36.92 | 38.46 | 40.08 | 41.59 | 42.52 |
| 18.7 | -0.02 | 0.06 | 34.83 | 35.61 | 36.95 | 38.49 | 40.10 | 41.61 | 42.54 |
| 18.8 | 0.00 | 0.06 | 34.86 | 35.64 | 36.97 | 38.52 | 40.13 | 41.63 | 42.56 |
| 18.9 | 0.01 | 0.06 | 34.88 | 35.66 | 37.00 | 38.54 | 40.15 | 41.66 | 42.58 |
| 19.0 | 0.03 | 0.06 | 34.91 | 35.69 | 37.03 | 38.57 | 40.17 | 41.68 | 42.60 |
| 19.1 | 0.05 | 0.06 | 34.93 | 35.71 | 37.05 | 38.60 | 40.20 | 41.70 | 42.62 |
| 19.2 | 0.07 | 0.06 | 34.96 | 35.74 | 37.08 | 38.62 | 40.22 | 41.72 | 42.64 |
| 19.3 | 0.09 | 0.06 | 34.98 | 35.76 | 37.10 | 38.65 | 40.25 | 41.74 | 42.66 |
| 19.4 | 0.11 | 0.06 | 35.01 | 35.79 | 37.13 | 38.67 | 40.27 | 41.76 | 42.68 |
| 19.5 | 0.13 | 0.06 | 35.03 | 35.81 | 37.16 | 38.70 | 40.29 | 41.78 | 42.69 |
| 19.6 | 0.15 | 0.06 | 35.05 | 35.84 | 37.18 | 38.72 | 40.32 | 41.80 | 42.71 |
| 19.7 | 0.17 | 0.06 | 35.08 | 35.86 | 37.21 | 38.75 | 40.34 | 41.82 | 42.73 |
| 19.8 | 0.19 | 0.06 | 35.10 | 35.89 | 37.23 | 38.77 | 40.36 | 41.84 | 42.75 |
| 19.9 | 0.21 | 0.06 | 35.13 | 35.91 | 37.26 | 38.80 | 40.39 | 41.86 | 42.77 |
