# Supplementary material for: Paediatric dominant and non-dominant handgrip reference curves and the association with body composition
Source: Ann Hum Biol. Author manuscript; Available in PMC 2024 Jun 10. (PMC11164034; doi:10.1080/03014460.2023.2298474)
Supplement: Supplemental Table 3 [file NIHMS1991062-supplement-Supplemental_Table_3.docx]

**Supplemental Table 3 Reference tables for dominant hand grip (A), non-dominant handgrip (B), and upper arm length (C) in females ages 6-19.9 years. Age in tenths of a year.**

1. Dominant handgrip, kg

|  | Females | | | | | | | | |
| --- | --- | --- | --- | --- | --- | --- | --- | --- | --- |
| Age, y | L | S | 5th | 10th | 25th | 50th (M) | 75th | 90th | 95th |
| 6.0 | -0.34 | 0.22 | 6.43 | 6.90 | 7.79 | 8.98 | 10.43 | 12.02 | 13.12 |
| 6.1 | -0.33 | 0.22 | 6.55 | 7.03 | 7.95 | 9.16 | 10.64 | 12.25 | 13.37 |
| 6.2 | -0.32 | 0.22 | 6.68 | 7.17 | 8.11 | 9.34 | 10.84 | 12.48 | 13.62 |
| 6.3 | -0.31 | 0.22 | 6.80 | 7.31 | 8.26 | 9.53 | 11.05 | 12.71 | 13.86 |
| 6.4 | -0.29 | 0.22 | 6.93 | 7.44 | 8.42 | 9.71 | 11.26 | 12.94 | 14.11 |
| 6.5 | -0.28 | 0.22 | 7.05 | 7.58 | 8.58 | 9.89 | 11.46 | 13.18 | 14.36 |
| 6.6 | -0.27 | 0.22 | 7.18 | 7.72 | 8.73 | 10.07 | 11.67 | 13.41 | 14.60 |
| 6.7 | -0.26 | 0.22 | 7.30 | 7.85 | 8.89 | 10.25 | 11.88 | 13.64 | 14.85 |
| 6.8 | -0.25 | 0.21 | 7.43 | 7.99 | 9.05 | 10.43 | 12.09 | 13.87 | 15.10 |
| 6.9 | -0.23 | 0.21 | 7.56 | 8.13 | 9.20 | 10.61 | 12.30 | 14.11 | 15.35 |
| 7.0 | -0.22 | 0.21 | 7.68 | 8.26 | 9.36 | 10.80 | 12.51 | 14.34 | 15.60 |
| 7.1 | -0.21 | 0.21 | 7.81 | 8.40 | 9.52 | 10.98 | 12.72 | 14.58 | 15.85 |
| 7.2 | -0.20 | 0.21 | 7.94 | 8.54 | 9.68 | 11.17 | 12.93 | 14.82 | 16.10 |
| 7.3 | -0.19 | 0.21 | 8.07 | 8.69 | 9.85 | 11.36 | 13.15 | 15.06 | 16.36 |
| 7.4 | -0.18 | 0.21 | 8.20 | 8.83 | 10.01 | 11.55 | 13.36 | 15.30 | 16.61 |
| 7.5 | -0.16 | 0.21 | 8.33 | 8.97 | 10.18 | 11.74 | 13.58 | 15.54 | 16.87 |
| 7.6 | -0.15 | 0.21 | 8.46 | 9.12 | 10.34 | 11.93 | 13.80 | 15.79 | 17.13 |
| 7.7 | -0.14 | 0.21 | 8.60 | 9.26 | 10.51 | 12.12 | 14.03 | 16.04 | 17.40 |
| 7.8 | -0.13 | 0.21 | 8.73 | 9.41 | 10.68 | 12.32 | 14.25 | 16.29 | 17.66 |
| 7.9 | -0.12 | 0.21 | 8.87 | 9.56 | 10.85 | 12.52 | 14.48 | 16.54 | 17.93 |
| 8.0 | -0.11 | 0.21 | 9.01 | 9.71 | 11.02 | 12.72 | 14.71 | 16.80 | 18.20 |
| 8.1 | -0.10 | 0.21 | 9.15 | 9.86 | 11.20 | 12.92 | 14.94 | 17.06 | 18.48 |
| 8.2 | -0.09 | 0.21 | 9.29 | 10.02 | 11.38 | 13.12 | 15.17 | 17.32 | 18.75 |
| 8.3 | -0.08 | 0.21 | 9.43 | 10.17 | 11.55 | 13.33 | 15.41 | 17.58 | 19.03 |
| 8.4 | -0.07 | 0.21 | 9.57 | 10.33 | 11.73 | 13.54 | 15.64 | 17.85 | 19.32 |
| 8.5 | -0.06 | 0.21 | 9.72 | 10.49 | 11.92 | 13.75 | 15.88 | 18.11 | 19.60 |
| 8.6 | -0.05 | 0.21 | 9.87 | 10.64 | 12.10 | 13.96 | 16.13 | 18.38 | 19.89 |
| 8.7 | -0.05 | 0.21 | 10.01 | 10.81 | 12.29 | 14.17 | 16.37 | 18.66 | 20.18 |
| 8.8 | -0.04 | 0.21 | 10.16 | 10.97 | 12.47 | 14.39 | 16.62 | 18.93 | 20.47 |
| 8.9 | -0.03 | 0.21 | 10.31 | 11.13 | 12.66 | 14.61 | 16.87 | 19.21 | 20.77 |
| 9.0 | -0.02 | 0.21 | 10.46 | 11.30 | 12.85 | 14.83 | 17.12 | 19.49 | 21.06 |
| 9.1 | -0.01 | 0.21 | 10.62 | 11.47 | 13.04 | 15.05 | 17.37 | 19.77 | 21.36 |
| 9.2 | 0.00 | 0.21 | 10.77 | 11.63 | 13.24 | 15.27 | 17.62 | 20.06 | 21.67 |
| 9.3 | 0.00 | 0.21 | 10.93 | 11.80 | 13.43 | 15.50 | 17.88 | 20.34 | 21.97 |
| 9.4 | 0.01 | 0.21 | 11.09 | 11.98 | 13.63 | 15.72 | 18.14 | 20.63 | 22.28 |
| 9.5 | 0.02 | 0.21 | 11.24 | 12.15 | 13.83 | 15.95 | 18.40 | 20.92 | 22.58 |
| 9.6 | 0.03 | 0.21 | 11.40 | 12.32 | 14.03 | 16.18 | 18.66 | 21.21 | 22.89 |
| 9.7 | 0.03 | 0.21 | 11.56 | 12.50 | 14.23 | 16.41 | 18.92 | 21.50 | 23.20 |
| 9.8 | 0.04 | 0.21 | 11.73 | 12.67 | 14.43 | 16.64 | 19.19 | 21.80 | 23.52 |
| 9.9 | 0.05 | 0.21 | 11.89 | 12.85 | 14.63 | 16.88 | 19.45 | 22.09 | 23.83 |
| 10.0 | 0.05 | 0.21 | 12.05 | 13.03 | 14.83 | 17.11 | 19.72 | 22.39 | 24.14 |
| 10.1 | 0.06 | 0.21 | 12.22 | 13.21 | 15.04 | 17.35 | 19.98 | 22.68 | 24.46 |
| 10.2 | 0.07 | 0.21 | 12.38 | 13.39 | 15.24 | 17.58 | 20.25 | 22.98 | 24.77 |
| 10.3 | 0.07 | 0.21 | 12.55 | 13.57 | 15.45 | 17.82 | 20.52 | 23.28 | 25.09 |
| 10.4 | 0.08 | 0.21 | 12.71 | 13.75 | 15.66 | 18.05 | 20.79 | 23.57 | 25.40 |
| 10.5 | 0.08 | 0.21 | 12.88 | 13.93 | 15.86 | 18.29 | 21.05 | 23.87 | 25.71 |
| 10.6 | 0.09 | 0.21 | 13.05 | 14.11 | 16.07 | 18.53 | 21.32 | 24.17 | 26.03 |
| 10.7 | 0.09 | 0.21 | 13.22 | 14.29 | 16.28 | 18.76 | 21.59 | 24.46 | 26.34 |
| 10.8 | 0.10 | 0.21 | 13.38 | 14.47 | 16.48 | 19.00 | 21.86 | 24.76 | 26.65 |
| 10.9 | 0.10 | 0.21 | 13.55 | 14.66 | 16.69 | 19.23 | 22.12 | 25.05 | 26.96 |
| 11.0 | 0.11 | 0.21 | 13.72 | 14.84 | 16.90 | 19.47 | 22.39 | 25.35 | 27.27 |
| 11.1 | 0.11 | 0.21 | 13.89 | 15.02 | 17.10 | 19.70 | 22.65 | 25.64 | 27.58 |
| 11.2 | 0.12 | 0.21 | 14.06 | 15.20 | 17.31 | 19.94 | 22.91 | 25.93 | 27.89 |
| 11.3 | 0.12 | 0.21 | 14.22 | 15.38 | 17.51 | 20.17 | 23.17 | 26.21 | 28.19 |
| 11.4 | 0.13 | 0.21 | 14.39 | 15.57 | 17.72 | 20.40 | 23.43 | 26.50 | 28.49 |
| 11.5 | 0.13 | 0.21 | 14.56 | 15.75 | 17.92 | 20.63 | 23.69 | 26.78 | 28.79 |
| 11.6 | 0.14 | 0.21 | 14.73 | 15.93 | 18.12 | 20.86 | 23.95 | 27.06 | 29.09 |
| 11.7 | 0.14 | 0.21 | 14.89 | 16.11 | 18.32 | 21.09 | 24.20 | 27.34 | 29.38 |
| 11.8 | 0.14 | 0.21 | 15.06 | 16.28 | 18.52 | 21.31 | 24.45 | 27.62 | 29.67 |
| 11.9 | 0.15 | 0.21 | 15.23 | 16.46 | 18.72 | 21.53 | 24.70 | 27.89 | 29.96 |
| 12.0 | 0.15 | 0.21 | 15.39 | 16.64 | 18.92 | 21.76 | 24.95 | 28.16 | 30.24 |
| 12.1 | 0.15 | 0.20 | 15.56 | 16.81 | 19.12 | 21.98 | 25.19 | 28.42 | 30.52 |
| 12.2 | 0.15 | 0.20 | 15.72 | 16.99 | 19.31 | 22.19 | 25.43 | 28.69 | 30.79 |
| 12.3 | 0.16 | 0.20 | 15.88 | 17.16 | 19.50 | 22.41 | 25.67 | 28.94 | 31.06 |
| 12.4 | 0.16 | 0.20 | 16.04 | 17.34 | 19.69 | 22.62 | 25.90 | 29.20 | 31.33 |
| 12.5 | 0.16 | 0.20 | 16.20 | 17.51 | 19.88 | 22.83 | 26.13 | 29.45 | 31.59 |
| 12.6 | 0.16 | 0.20 | 16.36 | 17.67 | 20.07 | 23.04 | 26.36 | 29.70 | 31.85 |
| 12.7 | 0.16 | 0.20 | 16.52 | 17.84 | 20.25 | 23.24 | 26.58 | 29.94 | 32.10 |
| 12.8 | 0.17 | 0.20 | 16.68 | 18.01 | 20.44 | 23.44 | 26.80 | 30.17 | 32.35 |
| 12.9 | 0.17 | 0.20 | 16.83 | 18.17 | 20.62 | 23.64 | 27.02 | 30.41 | 32.59 |
| 13.0 | 0.17 | 0.20 | 16.98 | 18.33 | 20.79 | 23.83 | 27.23 | 30.63 | 32.83 |
| 13.1 | 0.17 | 0.20 | 17.13 | 18.49 | 20.97 | 24.02 | 27.44 | 30.86 | 33.06 |
| 13.2 | 0.17 | 0.20 | 17.28 | 18.65 | 21.14 | 24.21 | 27.64 | 31.08 | 33.29 |
| 13.3 | 0.18 | 0.20 | 17.43 | 18.81 | 21.31 | 24.40 | 27.84 | 31.29 | 33.51 |
| 13.4 | 0.18 | 0.20 | 17.58 | 18.96 | 21.47 | 24.58 | 28.04 | 31.50 | 33.72 |
| 13.5 | 0.18 | 0.20 | 17.72 | 19.11 | 21.64 | 24.75 | 28.23 | 31.70 | 33.93 |
| 13.6 | 0.18 | 0.20 | 17.86 | 19.26 | 21.80 | 24.93 | 28.42 | 31.90 | 34.14 |
| 13.7 | 0.18 | 0.20 | 18.00 | 19.40 | 21.96 | 25.10 | 28.60 | 32.09 | 34.34 |
| 13.8 | 0.19 | 0.20 | 18.14 | 19.55 | 22.11 | 25.27 | 28.78 | 32.28 | 34.53 |
| 13.9 | 0.19 | 0.19 | 18.27 | 19.69 | 22.27 | 25.43 | 28.96 | 32.46 | 34.72 |
| 14.0 | 0.19 | 0.19 | 18.40 | 19.83 | 22.42 | 25.59 | 29.13 | 32.64 | 34.90 |
| 14.1 | 0.19 | 0.19 | 18.54 | 19.97 | 22.56 | 25.75 | 29.29 | 32.82 | 35.08 |
| 14.2 | 0.20 | 0.19 | 18.66 | 20.10 | 22.71 | 25.90 | 29.46 | 32.99 | 35.25 |
| 14.3 | 0.20 | 0.19 | 18.79 | 20.23 | 22.85 | 26.06 | 29.61 | 33.15 | 35.42 |
| 14.4 | 0.20 | 0.19 | 18.91 | 20.36 | 22.99 | 26.20 | 29.77 | 33.31 | 35.58 |
| 14.5 | 0.21 | 0.19 | 19.03 | 20.49 | 23.12 | 26.35 | 29.92 | 33.46 | 35.73 |
| 14.6 | 0.21 | 0.19 | 19.15 | 20.61 | 23.25 | 26.49 | 30.07 | 33.61 | 35.89 |
| 14.7 | 0.21 | 0.19 | 19.27 | 20.73 | 23.38 | 26.62 | 30.21 | 33.76 | 36.03 |
| 14.8 | 0.22 | 0.19 | 19.38 | 20.85 | 23.51 | 26.76 | 30.35 | 33.90 | 36.17 |
| 14.9 | 0.22 | 0.19 | 19.49 | 20.97 | 23.63 | 26.89 | 30.48 | 34.04 | 36.31 |
| 15.0 | 0.22 | 0.19 | 19.60 | 21.08 | 23.75 | 27.01 | 30.61 | 34.17 | 36.44 |
| 15.1 | 0.23 | 0.19 | 19.70 | 21.19 | 23.87 | 27.14 | 30.74 | 34.30 | 36.57 |
| 15.2 | 0.23 | 0.19 | 19.81 | 21.30 | 23.98 | 27.26 | 30.87 | 34.42 | 36.69 |
| 15.3 | 0.24 | 0.19 | 19.91 | 21.40 | 24.10 | 27.38 | 30.99 | 34.54 | 36.81 |
| 15.4 | 0.24 | 0.19 | 20.01 | 21.51 | 24.21 | 27.49 | 31.10 | 34.66 | 36.93 |
| 15.5 | 0.25 | 0.19 | 20.10 | 21.61 | 24.31 | 27.60 | 31.22 | 34.77 | 37.04 |
| 15.6 | 0.25 | 0.18 | 20.19 | 21.70 | 24.42 | 27.71 | 31.33 | 34.88 | 37.14 |
| 15.7 | 0.26 | 0.18 | 20.28 | 21.80 | 24.52 | 27.82 | 31.43 | 34.99 | 37.25 |
| 15.8 | 0.26 | 0.18 | 20.37 | 21.89 | 24.62 | 27.92 | 31.54 | 35.09 | 37.35 |
| 15.9 | 0.27 | 0.18 | 20.46 | 21.98 | 24.71 | 28.02 | 31.64 | 35.19 | 37.44 |
| 16.0 | 0.28 | 0.18 | 20.54 | 22.07 | 24.81 | 28.12 | 31.74 | 35.29 | 37.53 |
| 16.1 | 0.28 | 0.18 | 20.63 | 22.16 | 24.90 | 28.21 | 31.84 | 35.38 | 37.62 |
| 16.2 | 0.29 | 0.18 | 20.71 | 22.24 | 24.99 | 28.31 | 31.93 | 35.47 | 37.71 |
| 16.3 | 0.30 | 0.18 | 20.78 | 22.32 | 25.08 | 28.40 | 32.02 | 35.56 | 37.79 |
| 16.4 | 0.30 | 0.18 | 20.86 | 22.40 | 25.16 | 28.49 | 32.11 | 35.64 | 37.88 |
| 16.5 | 0.31 | 0.18 | 20.93 | 22.48 | 25.25 | 28.58 | 32.20 | 35.73 | 37.95 |
| 16.6 | 0.32 | 0.18 | 21.01 | 22.56 | 25.33 | 28.66 | 32.28 | 35.81 | 38.03 |
| 16.7 | 0.32 | 0.18 | 21.08 | 22.63 | 25.41 | 28.74 | 32.37 | 35.89 | 38.11 |
| 16.8 | 0.33 | 0.18 | 21.15 | 22.71 | 25.49 | 28.83 | 32.45 | 35.96 | 38.18 |
| 16.9 | 0.34 | 0.18 | 21.22 | 22.78 | 25.56 | 28.91 | 32.53 | 36.04 | 38.25 |
| 17.0 | 0.35 | 0.18 | 21.28 | 22.85 | 25.64 | 28.99 | 32.60 | 36.11 | 38.32 |
| 17.1 | 0.35 | 0.18 | 21.35 | 22.92 | 25.71 | 29.06 | 32.68 | 36.18 | 38.38 |
| 17.2 | 0.36 | 0.18 | 21.41 | 22.99 | 25.79 | 29.14 | 32.75 | 36.25 | 38.45 |
| 17.3 | 0.37 | 0.18 | 21.47 | 23.05 | 25.86 | 29.21 | 32.83 | 36.32 | 38.51 |
| 17.4 | 0.38 | 0.18 | 21.54 | 23.12 | 25.93 | 29.28 | 32.90 | 36.38 | 38.57 |
| 17.5 | 0.38 | 0.18 | 21.60 | 23.18 | 26.00 | 29.36 | 32.97 | 36.45 | 38.63 |
| 17.6 | 0.39 | 0.18 | 21.66 | 23.25 | 26.07 | 29.43 | 33.04 | 36.51 | 38.69 |
| 17.7 | 0.40 | 0.18 | 21.72 | 23.31 | 26.14 | 29.50 | 33.10 | 36.58 | 38.75 |
| 17.8 | 0.41 | 0.17 | 21.77 | 23.37 | 26.20 | 29.57 | 33.17 | 36.64 | 38.80 |
| 17.9 | 0.42 | 0.17 | 21.83 | 23.44 | 26.27 | 29.63 | 33.24 | 36.70 | 38.86 |
| 18.0 | 0.42 | 0.17 | 21.89 | 23.50 | 26.33 | 29.70 | 33.30 | 36.76 | 38.91 |
| 18.1 | 0.43 | 0.17 | 21.95 | 23.56 | 26.40 | 29.77 | 33.37 | 36.82 | 38.97 |
| 18.2 | 0.44 | 0.17 | 22.00 | 23.62 | 26.46 | 29.83 | 33.43 | 36.87 | 39.02 |
| 18.3 | 0.45 | 0.17 | 22.06 | 23.68 | 26.53 | 29.90 | 33.49 | 36.93 | 39.07 |
| 18.4 | 0.46 | 0.17 | 22.12 | 23.74 | 26.59 | 29.96 | 33.56 | 36.99 | 39.12 |
| 18.5 | 0.46 | 0.17 | 22.17 | 23.80 | 26.66 | 30.03 | 33.62 | 37.04 | 39.18 |
| 18.6 | 0.47 | 0.17 | 22.23 | 23.86 | 26.72 | 30.09 | 33.68 | 37.10 | 39.23 |
| 18.7 | 0.48 | 0.17 | 22.28 | 23.92 | 26.78 | 30.16 | 33.74 | 37.16 | 39.28 |
| 18.8 | 0.49 | 0.17 | 22.34 | 23.97 | 26.84 | 30.22 | 33.80 | 37.21 | 39.33 |
| 18.9 | 0.49 | 0.17 | 22.40 | 24.03 | 26.91 | 30.29 | 33.87 | 37.27 | 39.38 |
| 19.0 | 0.50 | 0.17 | 22.45 | 24.09 | 26.97 | 30.35 | 33.93 | 37.32 | 39.43 |
| 19.1 | 0.51 | 0.17 | 22.51 | 24.15 | 27.03 | 30.41 | 33.99 | 37.38 | 39.48 |
| 19.2 | 0.52 | 0.17 | 22.56 | 24.21 | 27.10 | 30.48 | 34.05 | 37.43 | 39.53 |
| 19.3 | 0.53 | 0.17 | 22.62 | 24.27 | 27.16 | 30.54 | 34.11 | 37.49 | 39.58 |
| 19.4 | 0.53 | 0.17 | 22.68 | 24.33 | 27.22 | 30.60 | 34.17 | 37.55 | 39.63 |
| 19.5 | 0.54 | 0.17 | 22.73 | 24.39 | 27.28 | 30.67 | 34.23 | 37.60 | 39.68 |
| 19.6 | 0.55 | 0.17 | 22.79 | 24.45 | 27.35 | 30.73 | 34.29 | 37.66 | 39.73 |
| 19.7 | 0.56 | 0.17 | 22.84 | 24.51 | 27.41 | 30.79 | 34.35 | 37.71 | 39.78 |
| 19.8 | 0.56 | 0.17 | 22.90 | 24.57 | 27.47 | 30.86 | 34.41 | 37.77 | 39.83 |
| 19.9 | 0.57 | 0.17 | 22.95 | 24.63 | 27.53 | 30.92 | 34.48 | 37.82 | 39.88 |

1. Non-dominant handgrip, kg

|  | Females | | | | | | | | |
| --- | --- | --- | --- | --- | --- | --- | --- | --- | --- |
| Age, y | L | S | 5th | 10th | 25th | 50th (M) | 75th | 90th | 95th |
| 6.0 | -0.51 | 0.22 | 6.28 | 6.73 | 7.58 | 8.74 | 10.19 | 11.83 | 13.01 |
| 6.1 | -0.49 | 0.22 | 6.40 | 6.85 | 7.73 | 8.91 | 10.38 | 12.05 | 13.24 |
| 6.2 | -0.48 | 0.22 | 6.51 | 6.98 | 7.87 | 9.08 | 10.58 | 12.26 | 13.47 |
| 6.3 | -0.46 | 0.22 | 6.63 | 7.10 | 8.02 | 9.24 | 10.77 | 12.48 | 13.69 |
| 6.4 | -0.44 | 0.22 | 6.74 | 7.23 | 8.16 | 9.41 | 10.96 | 12.69 | 13.92 |
| 6.5 | -0.43 | 0.22 | 6.86 | 7.35 | 8.30 | 9.58 | 11.15 | 12.91 | 14.15 |
| 6.6 | -0.41 | 0.22 | 6.97 | 7.48 | 8.45 | 9.75 | 11.34 | 13.12 | 14.38 |
| 6.7 | -0.39 | 0.22 | 7.09 | 7.60 | 8.59 | 9.91 | 11.54 | 13.34 | 14.60 |
| 6.8 | -0.38 | 0.22 | 7.20 | 7.73 | 8.74 | 10.08 | 11.73 | 13.55 | 14.83 |
| 6.9 | -0.36 | 0.22 | 7.32 | 7.85 | 8.88 | 10.25 | 11.92 | 13.77 | 15.06 |
| 7.0 | -0.35 | 0.22 | 7.43 | 7.98 | 9.03 | 10.42 | 12.12 | 13.98 | 15.28 |
| 7.1 | -0.33 | 0.22 | 7.55 | 8.11 | 9.17 | 10.59 | 12.31 | 14.20 | 15.51 |
| 7.2 | -0.31 | 0.22 | 7.66 | 8.23 | 9.32 | 10.76 | 12.51 | 14.42 | 15.74 |
| 7.3 | -0.30 | 0.22 | 7.78 | 8.36 | 9.47 | 10.93 | 12.71 | 14.64 | 15.97 |
| 7.4 | -0.28 | 0.22 | 7.89 | 8.49 | 9.62 | 11.11 | 12.90 | 14.86 | 16.21 |
| 7.5 | -0.27 | 0.22 | 8.01 | 8.62 | 9.77 | 11.28 | 13.10 | 15.08 | 16.44 |
| 7.6 | -0.25 | 0.22 | 8.13 | 8.75 | 9.92 | 11.46 | 13.30 | 15.30 | 16.67 |
| 7.7 | -0.24 | 0.22 | 8.25 | 8.88 | 10.07 | 11.63 | 13.51 | 15.53 | 16.91 |
| 7.8 | -0.22 | 0.22 | 8.37 | 9.01 | 10.22 | 11.81 | 13.71 | 15.75 | 17.15 |
| 7.9 | -0.21 | 0.22 | 8.49 | 9.14 | 10.38 | 11.99 | 13.92 | 15.98 | 17.39 |
| 8.0 | -0.19 | 0.22 | 8.61 | 9.28 | 10.53 | 12.17 | 14.12 | 16.21 | 17.64 |
| 8.1 | -0.18 | 0.22 | 8.73 | 9.41 | 10.69 | 12.35 | 14.33 | 16.44 | 17.88 |
| 8.2 | -0.16 | 0.22 | 8.86 | 9.55 | 10.85 | 12.54 | 14.54 | 16.68 | 18.13 |
| 8.3 | -0.15 | 0.22 | 8.98 | 9.68 | 11.01 | 12.72 | 14.76 | 16.92 | 18.38 |
| 8.4 | -0.14 | 0.22 | 9.11 | 9.82 | 11.17 | 12.91 | 14.97 | 17.15 | 18.63 |
| 8.5 | -0.12 | 0.22 | 9.23 | 9.96 | 11.33 | 13.10 | 15.19 | 17.40 | 18.88 |
| 8.6 | -0.11 | 0.22 | 9.36 | 10.10 | 11.50 | 13.29 | 15.41 | 17.64 | 19.14 |
| 8.7 | -0.10 | 0.22 | 9.49 | 10.25 | 11.66 | 13.48 | 15.63 | 17.88 | 19.40 |
| 8.8 | -0.08 | 0.22 | 9.62 | 10.39 | 11.83 | 13.68 | 15.85 | 18.13 | 19.66 |
| 8.9 | -0.07 | 0.22 | 9.75 | 10.53 | 12.00 | 13.88 | 16.07 | 18.38 | 19.93 |
| 9.0 | -0.06 | 0.22 | 9.88 | 10.68 | 12.17 | 14.07 | 16.30 | 18.63 | 20.19 |
| 9.1 | -0.05 | 0.22 | 10.02 | 10.83 | 12.34 | 14.27 | 16.53 | 18.89 | 20.46 |
| 9.2 | -0.04 | 0.22 | 10.15 | 10.97 | 12.51 | 14.47 | 16.76 | 19.14 | 20.73 |
| 9.3 | -0.02 | 0.22 | 10.29 | 11.12 | 12.68 | 14.68 | 16.99 | 19.40 | 21.00 |
| 9.4 | -0.01 | 0.22 | 10.43 | 11.27 | 12.86 | 14.88 | 17.22 | 19.66 | 21.27 |
| 9.5 | 0.00 | 0.22 | 10.56 | 11.43 | 13.04 | 15.09 | 17.46 | 19.92 | 21.55 |
| 9.6 | 0.01 | 0.22 | 10.70 | 11.58 | 13.21 | 15.29 | 17.69 | 20.18 | 21.83 |
| 9.7 | 0.02 | 0.22 | 10.84 | 11.73 | 13.39 | 15.50 | 17.93 | 20.44 | 22.11 |
| 9.8 | 0.03 | 0.22 | 10.98 | 11.89 | 13.57 | 15.71 | 18.17 | 20.71 | 22.39 |
| 9.9 | 0.04 | 0.22 | 11.12 | 12.04 | 13.75 | 15.92 | 18.41 | 20.97 | 22.67 |
| 10.0 | 0.05 | 0.22 | 11.26 | 12.20 | 13.93 | 16.13 | 18.65 | 21.24 | 22.95 |
| 10.1 | 0.06 | 0.22 | 11.41 | 12.36 | 14.12 | 16.34 | 18.89 | 21.51 | 23.23 |
| 10.2 | 0.07 | 0.22 | 11.55 | 12.51 | 14.30 | 16.55 | 19.13 | 21.78 | 23.51 |
| 10.3 | 0.08 | 0.22 | 11.69 | 12.67 | 14.48 | 16.76 | 19.37 | 22.04 | 23.80 |
| 10.4 | 0.09 | 0.22 | 11.84 | 12.83 | 14.67 | 16.98 | 19.62 | 22.31 | 24.08 |
| 10.5 | 0.10 | 0.22 | 11.98 | 12.99 | 14.85 | 17.19 | 19.86 | 22.58 | 24.36 |
| 10.6 | 0.11 | 0.22 | 12.13 | 13.15 | 15.03 | 17.40 | 20.10 | 22.85 | 24.65 |
| 10.7 | 0.11 | 0.22 | 12.27 | 13.31 | 15.22 | 17.62 | 20.34 | 23.12 | 24.93 |
| 10.8 | 0.12 | 0.22 | 12.42 | 13.47 | 15.40 | 17.83 | 20.58 | 23.38 | 25.21 |
| 10.9 | 0.13 | 0.21 | 12.56 | 13.63 | 15.59 | 18.04 | 20.82 | 23.65 | 25.49 |
| 11.0 | 0.14 | 0.21 | 12.71 | 13.79 | 15.77 | 18.25 | 21.06 | 23.91 | 25.77 |
| 11.1 | 0.15 | 0.21 | 12.85 | 13.94 | 15.95 | 18.46 | 21.30 | 24.18 | 26.05 |
| 11.2 | 0.15 | 0.21 | 13.00 | 14.10 | 16.14 | 18.67 | 21.54 | 24.44 | 26.32 |
| 11.3 | 0.16 | 0.21 | 13.14 | 14.26 | 16.32 | 18.88 | 21.78 | 24.70 | 26.60 |
| 11.4 | 0.17 | 0.21 | 13.29 | 14.42 | 16.50 | 19.09 | 22.01 | 24.96 | 26.87 |
| 11.5 | 0.17 | 0.21 | 13.43 | 14.58 | 16.68 | 19.30 | 22.25 | 25.22 | 27.14 |
| 11.6 | 0.18 | 0.21 | 13.58 | 14.74 | 16.86 | 19.51 | 22.48 | 25.47 | 27.41 |
| 11.7 | 0.18 | 0.21 | 13.72 | 14.89 | 17.04 | 19.71 | 22.71 | 25.73 | 27.68 |
| 11.8 | 0.19 | 0.21 | 13.86 | 15.05 | 17.22 | 19.91 | 22.94 | 25.98 | 27.94 |
| 11.9 | 0.20 | 0.21 | 14.01 | 15.21 | 17.40 | 20.12 | 23.17 | 26.23 | 28.20 |
| 12.0 | 0.20 | 0.21 | 14.15 | 15.36 | 17.57 | 20.31 | 23.39 | 26.47 | 28.46 |
| 12.1 | 0.20 | 0.21 | 14.29 | 15.52 | 17.75 | 20.51 | 23.61 | 26.71 | 28.72 |
| 12.2 | 0.21 | 0.21 | 14.43 | 15.67 | 17.92 | 20.71 | 23.83 | 26.95 | 28.97 |
| 12.3 | 0.21 | 0.21 | 14.58 | 15.82 | 18.09 | 20.90 | 24.05 | 27.19 | 29.22 |
| 12.4 | 0.22 | 0.21 | 14.72 | 15.97 | 18.26 | 21.09 | 24.26 | 27.42 | 29.46 |
| 12.5 | 0.22 | 0.21 | 14.85 | 16.12 | 18.43 | 21.28 | 24.47 | 27.65 | 29.70 |
| 12.6 | 0.22 | 0.21 | 14.99 | 16.27 | 18.60 | 21.47 | 24.68 | 27.87 | 29.93 |
| 12.7 | 0.23 | 0.21 | 15.13 | 16.42 | 18.76 | 21.65 | 24.88 | 28.09 | 30.16 |
| 12.8 | 0.23 | 0.21 | 15.26 | 16.56 | 18.92 | 21.83 | 25.08 | 28.31 | 30.39 |
| 12.9 | 0.23 | 0.21 | 15.40 | 16.70 | 19.08 | 22.01 | 25.27 | 28.52 | 30.61 |
| 13.0 | 0.24 | 0.21 | 15.53 | 16.85 | 19.24 | 22.19 | 25.47 | 28.73 | 30.83 |
| 13.1 | 0.24 | 0.21 | 15.66 | 16.99 | 19.39 | 22.36 | 25.66 | 28.93 | 31.04 |
| 13.2 | 0.24 | 0.21 | 15.79 | 17.12 | 19.55 | 22.53 | 25.84 | 29.13 | 31.24 |
| 13.3 | 0.25 | 0.21 | 15.92 | 17.26 | 19.70 | 22.69 | 26.02 | 29.33 | 31.45 |
| 13.4 | 0.25 | 0.21 | 16.04 | 17.39 | 19.85 | 22.86 | 26.20 | 29.52 | 31.64 |
| 13.5 | 0.25 | 0.21 | 16.17 | 17.52 | 19.99 | 23.02 | 26.37 | 29.70 | 31.83 |
| 13.6 | 0.26 | 0.20 | 16.29 | 17.65 | 20.13 | 23.17 | 26.54 | 29.88 | 32.02 |
| 13.7 | 0.26 | 0.20 | 16.41 | 17.78 | 20.27 | 23.33 | 26.71 | 30.06 | 32.20 |
| 13.8 | 0.26 | 0.20 | 16.53 | 17.91 | 20.41 | 23.48 | 26.87 | 30.23 | 32.38 |
| 13.9 | 0.26 | 0.20 | 16.64 | 18.03 | 20.55 | 23.63 | 27.03 | 30.40 | 32.55 |
| 14.0 | 0.27 | 0.20 | 16.76 | 18.15 | 20.68 | 23.77 | 27.19 | 30.56 | 32.72 |
| 14.1 | 0.27 | 0.20 | 16.87 | 18.27 | 20.81 | 23.91 | 27.34 | 30.72 | 32.88 |
| 14.2 | 0.27 | 0.20 | 16.98 | 18.39 | 20.94 | 24.05 | 27.49 | 30.88 | 33.04 |
| 14.3 | 0.28 | 0.20 | 17.09 | 18.51 | 21.06 | 24.19 | 27.63 | 31.03 | 33.20 |
| 14.4 | 0.28 | 0.20 | 17.20 | 18.62 | 21.19 | 24.32 | 27.77 | 31.17 | 33.34 |
| 14.5 | 0.28 | 0.20 | 17.31 | 18.73 | 21.31 | 24.45 | 27.91 | 31.32 | 33.49 |
| 14.6 | 0.29 | 0.20 | 17.41 | 18.84 | 21.43 | 24.57 | 28.04 | 31.46 | 33.63 |
| 14.7 | 0.29 | 0.20 | 17.51 | 18.95 | 21.54 | 24.70 | 28.17 | 31.59 | 33.77 |
| 14.8 | 0.29 | 0.20 | 17.61 | 19.05 | 21.65 | 24.82 | 28.30 | 31.72 | 33.90 |
| 14.9 | 0.30 | 0.20 | 17.71 | 19.15 | 21.76 | 24.94 | 28.42 | 31.85 | 34.02 |
| 15.0 | 0.30 | 0.20 | 17.80 | 19.25 | 21.87 | 25.05 | 28.54 | 31.97 | 34.15 |
| 15.1 | 0.31 | 0.20 | 17.89 | 19.35 | 21.98 | 25.16 | 28.66 | 32.09 | 34.27 |
| 15.2 | 0.31 | 0.20 | 17.98 | 19.45 | 22.08 | 25.27 | 28.77 | 32.20 | 34.38 |
| 15.3 | 0.31 | 0.20 | 18.07 | 19.54 | 22.18 | 25.38 | 28.88 | 32.32 | 34.50 |
| 15.4 | 0.32 | 0.20 | 18.16 | 19.63 | 22.28 | 25.48 | 28.99 | 32.43 | 34.60 |
| 15.5 | 0.32 | 0.19 | 18.24 | 19.72 | 22.37 | 25.58 | 29.10 | 32.53 | 34.71 |
| 15.6 | 0.33 | 0.19 | 18.32 | 19.80 | 22.46 | 25.68 | 29.20 | 32.63 | 34.81 |
| 15.7 | 0.33 | 0.19 | 18.40 | 19.89 | 22.56 | 25.78 | 29.30 | 32.73 | 34.91 |
| 15.8 | 0.34 | 0.19 | 18.48 | 19.97 | 22.64 | 25.87 | 29.39 | 32.83 | 35.00 |
| 15.9 | 0.34 | 0.19 | 18.56 | 20.05 | 22.73 | 25.96 | 29.49 | 32.92 | 35.09 |
| 16.0 | 0.35 | 0.19 | 18.63 | 20.13 | 22.82 | 26.05 | 29.58 | 33.01 | 35.18 |
| 16.1 | 0.35 | 0.19 | 18.70 | 20.21 | 22.90 | 26.14 | 29.67 | 33.10 | 35.27 |
| 16.2 | 0.36 | 0.19 | 18.77 | 20.28 | 22.98 | 26.23 | 29.76 | 33.19 | 35.36 |
| 16.3 | 0.36 | 0.19 | 18.84 | 20.35 | 23.06 | 26.31 | 29.84 | 33.28 | 35.44 |
| 16.4 | 0.37 | 0.19 | 18.91 | 20.43 | 23.14 | 26.39 | 29.93 | 33.36 | 35.52 |
| 16.5 | 0.37 | 0.19 | 18.97 | 20.49 | 23.21 | 26.47 | 30.01 | 33.44 | 35.60 |
| 16.6 | 0.38 | 0.19 | 19.04 | 20.56 | 23.29 | 26.55 | 30.09 | 33.52 | 35.67 |
| 16.7 | 0.39 | 0.19 | 19.10 | 20.63 | 23.36 | 26.63 | 30.17 | 33.59 | 35.75 |
| 16.8 | 0.39 | 0.19 | 19.16 | 20.69 | 23.43 | 26.70 | 30.24 | 33.67 | 35.82 |
| 16.9 | 0.40 | 0.19 | 19.22 | 20.76 | 23.50 | 26.78 | 30.32 | 33.74 | 35.89 |
| 17.0 | 0.40 | 0.19 | 19.27 | 20.82 | 23.56 | 26.85 | 30.39 | 33.81 | 35.96 |
| 17.1 | 0.41 | 0.19 | 19.33 | 20.88 | 23.63 | 26.92 | 30.46 | 33.88 | 36.03 |
| 17.2 | 0.42 | 0.19 | 19.39 | 20.94 | 23.70 | 26.99 | 30.53 | 33.95 | 36.09 |
| 17.3 | 0.42 | 0.19 | 19.44 | 21.00 | 23.76 | 27.06 | 30.60 | 34.02 | 36.16 |
| 17.4 | 0.43 | 0.19 | 19.49 | 21.05 | 23.82 | 27.12 | 30.67 | 34.08 | 36.22 |
| 17.5 | 0.44 | 0.19 | 19.54 | 21.11 | 23.88 | 27.19 | 30.73 | 34.15 | 36.28 |
| 17.6 | 0.44 | 0.19 | 19.59 | 21.16 | 23.94 | 27.25 | 30.80 | 34.21 | 36.34 |
| 17.7 | 0.45 | 0.19 | 19.64 | 21.22 | 24.00 | 27.31 | 30.86 | 34.27 | 36.40 |
| 17.8 | 0.46 | 0.19 | 19.69 | 21.27 | 24.06 | 27.38 | 30.93 | 34.33 | 36.46 |
| 17.9 | 0.46 | 0.19 | 19.74 | 21.32 | 24.12 | 27.44 | 30.99 | 34.39 | 36.51 |
| 18.0 | 0.47 | 0.19 | 19.78 | 21.37 | 24.18 | 27.50 | 31.05 | 34.45 | 36.57 |
| 18.1 | 0.48 | 0.19 | 19.83 | 21.42 | 24.23 | 27.56 | 31.11 | 34.51 | 36.62 |
| 18.2 | 0.48 | 0.18 | 19.88 | 21.47 | 24.29 | 27.62 | 31.17 | 34.56 | 36.68 |
| 18.3 | 0.49 | 0.18 | 19.92 | 21.52 | 24.34 | 27.68 | 31.23 | 34.62 | 36.73 |
| 18.4 | 0.50 | 0.18 | 19.97 | 21.57 | 24.40 | 27.73 | 31.29 | 34.68 | 36.78 |
| 18.5 | 0.50 | 0.18 | 20.01 | 21.62 | 24.45 | 27.79 | 31.34 | 34.73 | 36.83 |
| 18.6 | 0.51 | 0.18 | 20.06 | 21.67 | 24.50 | 27.85 | 31.40 | 34.78 | 36.89 |
| 18.7 | 0.52 | 0.18 | 20.10 | 21.72 | 24.56 | 27.90 | 31.46 | 34.84 | 36.94 |
| 18.8 | 0.52 | 0.18 | 20.14 | 21.76 | 24.61 | 27.96 | 31.51 | 34.89 | 36.99 |
| 18.9 | 0.53 | 0.18 | 20.19 | 21.81 | 24.66 | 28.02 | 31.57 | 34.95 | 37.04 |
| 19.0 | 0.54 | 0.18 | 20.23 | 21.86 | 24.72 | 28.07 | 31.63 | 35.00 | 37.09 |
| 19.1 | 0.55 | 0.18 | 20.27 | 21.91 | 24.77 | 28.13 | 31.68 | 35.05 | 37.14 |
| 19.2 | 0.55 | 0.18 | 20.32 | 21.95 | 24.82 | 28.18 | 31.74 | 35.10 | 37.19 |
| 19.3 | 0.56 | 0.18 | 20.36 | 22.00 | 24.87 | 28.24 | 31.79 | 35.16 | 37.24 |
| 19.4 | 0.57 | 0.18 | 20.40 | 22.05 | 24.93 | 28.30 | 31.85 | 35.21 | 37.29 |
| 19.5 | 0.57 | 0.18 | 20.44 | 22.09 | 24.98 | 28.35 | 31.90 | 35.26 | 37.34 |
| 19.6 | 0.58 | 0.18 | 20.49 | 22.14 | 25.03 | 28.41 | 31.96 | 35.31 | 37.38 |
| 19.7 | 0.59 | 0.18 | 20.53 | 22.19 | 25.08 | 28.46 | 32.02 | 35.37 | 37.43 |
| 19.8 | 0.59 | 0.18 | 20.57 | 22.24 | 25.14 | 28.52 | 32.07 | 35.42 | 37.48 |
| 19.9 | 0.60 | 0.18 | 20.62 | 22.28 | 25.19 | 28.57 | 32.13 | 35.47 | 37.53 |

1. Upper arm length, cm

|  | Females | | | | | | | | |
| --- | --- | --- | --- | --- | --- | --- | --- | --- | --- |
| Age, y | L | S | 5th | 10th | 25th | 50th (M) | 75th | 90th | 95th |
| 6.0 | -0.82 | 0.07 | 21.41 | 21.91 | 22.79 | 23.86 | 25.01 | 26.15 | 26.87 |
| 6.1 | -0.82 | 0.07 | 21.55 | 22.05 | 22.94 | 24.01 | 25.18 | 26.32 | 27.05 |
| 6.2 | -0.82 | 0.07 | 21.69 | 22.20 | 23.09 | 24.17 | 25.34 | 26.49 | 27.22 |
| 6.3 | -0.82 | 0.07 | 21.83 | 22.34 | 23.25 | 24.33 | 25.51 | 26.66 | 27.40 |
| 6.4 | -0.82 | 0.07 | 21.97 | 22.49 | 23.40 | 24.49 | 25.67 | 26.84 | 27.58 |
| 6.5 | -0.82 | 0.07 | 22.11 | 22.63 | 23.55 | 24.64 | 25.84 | 27.01 | 27.75 |
| 6.6 | -0.82 | 0.07 | 22.26 | 22.78 | 23.70 | 24.80 | 26.00 | 27.18 | 27.93 |
| 6.7 | -0.82 | 0.07 | 22.40 | 22.92 | 23.85 | 24.96 | 26.17 | 27.35 | 28.11 |
| 6.8 | -0.82 | 0.07 | 22.54 | 23.07 | 24.00 | 25.12 | 26.33 | 27.53 | 28.29 |
| 6.9 | -0.82 | 0.07 | 22.68 | 23.21 | 24.15 | 25.27 | 26.50 | 27.70 | 28.46 |
| 7.0 | -0.82 | 0.07 | 22.82 | 23.36 | 24.30 | 25.43 | 26.66 | 27.87 | 28.64 |
| 7.1 | -0.82 | 0.07 | 22.96 | 23.50 | 24.45 | 25.59 | 26.83 | 28.04 | 28.82 |
| 7.2 | -0.82 | 0.07 | 23.11 | 23.65 | 24.60 | 25.75 | 26.99 | 28.22 | 28.99 |
| 7.3 | -0.82 | 0.07 | 23.25 | 23.79 | 24.75 | 25.91 | 27.16 | 28.39 | 29.17 |
| 7.4 | -0.82 | 0.07 | 23.39 | 23.94 | 24.90 | 26.06 | 27.32 | 28.56 | 29.35 |
| 7.5 | -0.82 | 0.07 | 23.53 | 24.08 | 25.06 | 26.22 | 27.49 | 28.73 | 29.52 |
| 7.6 | -0.82 | 0.07 | 23.68 | 24.23 | 25.21 | 26.38 | 27.66 | 28.91 | 29.70 |
| 7.7 | -0.81 | 0.07 | 23.82 | 24.37 | 25.36 | 26.54 | 27.82 | 29.08 | 29.88 |
| 7.8 | -0.81 | 0.07 | 23.96 | 24.52 | 25.51 | 26.70 | 27.99 | 29.25 | 30.06 |
| 7.9 | -0.81 | 0.07 | 24.10 | 24.66 | 25.66 | 26.85 | 28.15 | 29.42 | 30.23 |
| 8.0 | -0.81 | 0.07 | 24.25 | 24.81 | 25.81 | 27.01 | 28.32 | 29.59 | 30.41 |
| 8.1 | -0.81 | 0.07 | 24.39 | 24.96 | 25.96 | 27.17 | 28.48 | 29.77 | 30.58 |
| 8.2 | -0.80 | 0.07 | 24.53 | 25.10 | 26.11 | 27.33 | 28.65 | 29.94 | 30.76 |
| 8.3 | -0.80 | 0.07 | 24.67 | 25.25 | 26.27 | 27.48 | 28.81 | 30.11 | 30.93 |
| 8.4 | -0.80 | 0.07 | 24.81 | 25.39 | 26.42 | 27.64 | 28.97 | 30.28 | 31.11 |
| 8.5 | -0.79 | 0.07 | 24.95 | 25.54 | 26.57 | 27.80 | 29.14 | 30.45 | 31.28 |
| 8.6 | -0.79 | 0.07 | 25.10 | 25.68 | 26.72 | 27.96 | 29.30 | 30.62 | 31.46 |
| 8.7 | -0.78 | 0.07 | 25.24 | 25.83 | 26.87 | 28.11 | 29.47 | 30.79 | 31.63 |
| 8.8 | -0.78 | 0.07 | 25.38 | 25.97 | 27.02 | 28.27 | 29.63 | 30.96 | 31.80 |
| 8.9 | -0.77 | 0.07 | 25.52 | 26.11 | 27.17 | 28.42 | 29.79 | 31.13 | 31.97 |
| 9.0 | -0.76 | 0.07 | 25.66 | 26.26 | 27.31 | 28.58 | 29.95 | 31.29 | 32.15 |
| 9.1 | -0.76 | 0.07 | 25.80 | 26.40 | 27.46 | 28.73 | 30.11 | 31.46 | 32.32 |
| 9.2 | -0.75 | 0.07 | 25.93 | 26.54 | 27.61 | 28.89 | 30.27 | 31.63 | 32.48 |
| 9.3 | -0.74 | 0.07 | 26.07 | 26.68 | 27.76 | 29.04 | 30.43 | 31.79 | 32.65 |
| 9.4 | -0.73 | 0.07 | 26.21 | 26.82 | 27.90 | 29.19 | 30.59 | 31.95 | 32.82 |
| 9.5 | -0.72 | 0.07 | 26.35 | 26.96 | 28.05 | 29.35 | 30.75 | 32.12 | 32.99 |
| 9.6 | -0.71 | 0.07 | 26.48 | 27.10 | 28.19 | 29.50 | 30.91 | 32.28 | 33.15 |
| 9.7 | -0.70 | 0.07 | 26.62 | 27.24 | 28.34 | 29.65 | 31.06 | 32.44 | 33.31 |
| 9.8 | -0.69 | 0.07 | 26.75 | 27.37 | 28.48 | 29.79 | 31.22 | 32.60 | 33.48 |
| 9.9 | -0.67 | 0.07 | 26.88 | 27.51 | 28.62 | 29.94 | 31.37 | 32.76 | 33.64 |
| 10.0 | -0.66 | 0.07 | 27.01 | 27.65 | 28.76 | 30.09 | 31.52 | 32.91 | 33.79 |
| 10.1 | -0.65 | 0.07 | 27.14 | 27.78 | 28.90 | 30.23 | 31.67 | 33.07 | 33.95 |
| 10.2 | -0.63 | 0.07 | 27.27 | 27.91 | 29.04 | 30.38 | 31.82 | 33.22 | 34.11 |
| 10.3 | -0.62 | 0.07 | 27.40 | 28.04 | 29.18 | 30.52 | 31.97 | 33.37 | 34.26 |
| 10.4 | -0.61 | 0.07 | 27.53 | 28.17 | 29.31 | 30.66 | 32.11 | 33.52 | 34.41 |
| 10.5 | -0.59 | 0.07 | 27.65 | 28.30 | 29.45 | 30.80 | 32.26 | 33.67 | 34.56 |
| 10.6 | -0.57 | 0.07 | 27.78 | 28.43 | 29.58 | 30.94 | 32.40 | 33.82 | 34.71 |
| 10.7 | -0.56 | 0.07 | 27.90 | 28.56 | 29.71 | 31.08 | 32.54 | 33.96 | 34.85 |
| 10.8 | -0.54 | 0.07 | 28.02 | 28.68 | 29.84 | 31.21 | 32.68 | 34.10 | 35.00 |
| 10.9 | -0.52 | 0.07 | 28.14 | 28.80 | 29.97 | 31.34 | 32.82 | 34.24 | 35.14 |
| 11.0 | -0.51 | 0.07 | 28.26 | 28.92 | 30.09 | 31.47 | 32.95 | 34.38 | 35.28 |
| 11.1 | -0.49 | 0.07 | 28.38 | 29.04 | 30.22 | 31.60 | 33.09 | 34.51 | 35.41 |
| 11.2 | -0.47 | 0.07 | 28.49 | 29.16 | 30.34 | 31.73 | 33.22 | 34.65 | 35.54 |
| 11.3 | -0.45 | 0.07 | 28.60 | 29.28 | 30.46 | 31.86 | 33.35 | 34.78 | 35.68 |
| 11.4 | -0.44 | 0.07 | 28.71 | 29.39 | 30.58 | 31.98 | 33.47 | 34.91 | 35.80 |
| 11.5 | -0.42 | 0.07 | 28.82 | 29.50 | 30.70 | 32.10 | 33.60 | 35.03 | 35.93 |
| 11.6 | -0.40 | 0.07 | 28.93 | 29.62 | 30.81 | 32.22 | 33.72 | 35.15 | 36.05 |
| 11.7 | -0.38 | 0.07 | 29.04 | 29.72 | 30.92 | 32.34 | 33.84 | 35.27 | 36.17 |
| 11.8 | -0.36 | 0.07 | 29.14 | 29.83 | 31.04 | 32.45 | 33.95 | 35.39 | 36.29 |
| 11.9 | -0.34 | 0.07 | 29.24 | 29.94 | 31.14 | 32.56 | 34.07 | 35.51 | 36.41 |
| 12.0 | -0.33 | 0.07 | 29.34 | 30.04 | 31.25 | 32.67 | 34.18 | 35.62 | 36.52 |
| 12.1 | -0.31 | 0.07 | 29.44 | 30.14 | 31.35 | 32.78 | 34.29 | 35.73 | 36.63 |
| 12.2 | -0.29 | 0.07 | 29.54 | 30.24 | 31.46 | 32.88 | 34.40 | 35.84 | 36.74 |
| 12.3 | -0.27 | 0.07 | 29.63 | 30.33 | 31.56 | 32.99 | 34.50 | 35.94 | 36.84 |
| 12.4 | -0.25 | 0.07 | 29.72 | 30.43 | 31.65 | 33.09 | 34.60 | 36.04 | 36.94 |
| 12.5 | -0.23 | 0.07 | 29.81 | 30.52 | 31.75 | 33.19 | 34.70 | 36.14 | 37.04 |
| 12.6 | -0.21 | 0.07 | 29.90 | 30.61 | 31.84 | 33.28 | 34.80 | 36.24 | 37.14 |
| 12.7 | -0.19 | 0.07 | 29.98 | 30.70 | 31.93 | 33.37 | 34.89 | 36.33 | 37.23 |
| 12.8 | -0.17 | 0.07 | 30.07 | 30.78 | 32.02 | 33.46 | 34.98 | 36.43 | 37.32 |
| 12.9 | -0.16 | 0.07 | 30.15 | 30.86 | 32.10 | 33.55 | 35.07 | 36.51 | 37.41 |
| 13.0 | -0.14 | 0.07 | 30.22 | 30.94 | 32.19 | 33.64 | 35.16 | 36.60 | 37.49 |
| 13.1 | -0.12 | 0.07 | 30.30 | 31.02 | 32.27 | 33.72 | 35.24 | 36.68 | 37.57 |
| 13.2 | -0.10 | 0.07 | 30.37 | 31.09 | 32.35 | 33.80 | 35.32 | 36.76 | 37.65 |
| 13.3 | -0.08 | 0.07 | 30.44 | 31.17 | 32.42 | 33.88 | 35.40 | 36.84 | 37.73 |
| 13.4 | -0.06 | 0.07 | 30.51 | 31.24 | 32.49 | 33.95 | 35.47 | 36.91 | 37.80 |
| 13.5 | -0.04 | 0.07 | 30.58 | 31.31 | 32.56 | 34.02 | 35.55 | 36.98 | 37.87 |
| 13.6 | -0.02 | 0.06 | 30.64 | 31.37 | 32.63 | 34.09 | 35.62 | 37.05 | 37.94 |
| 13.7 | 0.00 | 0.06 | 30.70 | 31.44 | 32.70 | 34.16 | 35.68 | 37.12 | 38.00 |
| 13.8 | 0.02 | 0.06 | 30.76 | 31.50 | 32.76 | 34.22 | 35.75 | 37.18 | 38.07 |
| 13.9 | 0.03 | 0.06 | 30.82 | 31.56 | 32.82 | 34.29 | 35.81 | 37.25 | 38.13 |
| 14.0 | 0.05 | 0.06 | 30.88 | 31.61 | 32.88 | 34.35 | 35.87 | 37.30 | 38.18 |
| 14.1 | 0.07 | 0.06 | 30.93 | 31.67 | 32.94 | 34.40 | 35.93 | 37.36 | 38.24 |
| 14.2 | 0.09 | 0.06 | 30.98 | 31.72 | 32.99 | 34.46 | 35.99 | 37.42 | 38.29 |
| 14.3 | 0.11 | 0.06 | 31.03 | 31.77 | 33.05 | 34.51 | 36.04 | 37.47 | 38.34 |
| 14.4 | 0.12 | 0.06 | 31.08 | 31.82 | 33.10 | 34.57 | 36.09 | 37.52 | 38.39 |
| 14.5 | 0.14 | 0.06 | 31.12 | 31.86 | 33.14 | 34.61 | 36.14 | 37.57 | 38.44 |
| 14.6 | 0.16 | 0.06 | 31.16 | 31.91 | 33.19 | 34.66 | 36.19 | 37.61 | 38.48 |
| 14.7 | 0.18 | 0.06 | 31.20 | 31.95 | 33.23 | 34.71 | 36.23 | 37.65 | 38.53 |
| 14.8 | 0.19 | 0.06 | 31.24 | 31.99 | 33.28 | 34.75 | 36.27 | 37.70 | 38.57 |
| 14.9 | 0.21 | 0.06 | 31.28 | 32.03 | 33.32 | 34.79 | 36.32 | 37.74 | 38.60 |
| 15.0 | 0.23 | 0.06 | 31.31 | 32.07 | 33.35 | 34.83 | 36.35 | 37.77 | 38.64 |
| 15.1 | 0.25 | 0.06 | 31.35 | 32.10 | 33.39 | 34.87 | 36.39 | 37.81 | 38.68 |
| 15.2 | 0.26 | 0.06 | 31.38 | 32.13 | 33.43 | 34.90 | 36.43 | 37.84 | 38.71 |
| 15.3 | 0.28 | 0.06 | 31.41 | 32.16 | 33.46 | 34.94 | 36.46 | 37.88 | 38.74 |
| 15.4 | 0.29 | 0.06 | 31.44 | 32.19 | 33.49 | 34.97 | 36.49 | 37.91 | 38.77 |
| 15.5 | 0.31 | 0.06 | 31.47 | 32.22 | 33.52 | 35.00 | 36.52 | 37.94 | 38.80 |
| 15.6 | 0.33 | 0.06 | 31.49 | 32.25 | 33.55 | 35.03 | 36.55 | 37.96 | 38.82 |
| 15.7 | 0.34 | 0.06 | 31.52 | 32.28 | 33.58 | 35.06 | 36.58 | 37.99 | 38.85 |
| 15.8 | 0.36 | 0.06 | 31.54 | 32.30 | 33.60 | 35.08 | 36.61 | 38.02 | 38.87 |
| 15.9 | 0.37 | 0.06 | 31.56 | 32.32 | 33.63 | 35.11 | 36.63 | 38.04 | 38.90 |
| 16.0 | 0.39 | 0.06 | 31.58 | 32.35 | 33.65 | 35.13 | 36.66 | 38.06 | 38.92 |
| 16.1 | 0.40 | 0.06 | 31.60 | 32.37 | 33.67 | 35.16 | 36.68 | 38.09 | 38.94 |
| 16.2 | 0.42 | 0.06 | 31.62 | 32.39 | 33.70 | 35.18 | 36.70 | 38.11 | 38.96 |
| 16.3 | 0.43 | 0.06 | 31.64 | 32.41 | 33.72 | 35.20 | 36.72 | 38.13 | 38.98 |
| 16.4 | 0.45 | 0.06 | 31.66 | 32.43 | 33.74 | 35.22 | 36.74 | 38.15 | 39.00 |
| 16.5 | 0.46 | 0.06 | 31.68 | 32.45 | 33.76 | 35.24 | 36.76 | 38.16 | 39.01 |
| 16.6 | 0.48 | 0.06 | 31.69 | 32.46 | 33.78 | 35.26 | 36.78 | 38.18 | 39.03 |
| 16.7 | 0.49 | 0.06 | 31.71 | 32.48 | 33.79 | 35.28 | 36.80 | 38.20 | 39.05 |
| 16.8 | 0.50 | 0.06 | 31.72 | 32.50 | 33.81 | 35.30 | 36.82 | 38.21 | 39.06 |
| 16.9 | 0.52 | 0.06 | 31.74 | 32.51 | 33.83 | 35.32 | 36.83 | 38.23 | 39.08 |
| 17.0 | 0.53 | 0.06 | 31.75 | 32.53 | 33.84 | 35.33 | 36.85 | 38.25 | 39.09 |
| 17.1 | 0.55 | 0.06 | 31.77 | 32.54 | 33.86 | 35.35 | 36.87 | 38.26 | 39.10 |
| 17.2 | 0.56 | 0.06 | 31.78 | 32.56 | 33.88 | 35.36 | 36.88 | 38.27 | 39.12 |
| 17.3 | 0.58 | 0.06 | 31.79 | 32.57 | 33.89 | 35.38 | 36.90 | 38.29 | 39.13 |
| 17.4 | 0.59 | 0.06 | 31.80 | 32.58 | 33.90 | 35.39 | 36.91 | 38.30 | 39.14 |
| 17.5 | 0.61 | 0.06 | 31.82 | 32.60 | 33.92 | 35.41 | 36.92 | 38.31 | 39.15 |
| 17.6 | 0.62 | 0.06 | 31.83 | 32.61 | 33.93 | 35.42 | 36.94 | 38.33 | 39.16 |
| 17.7 | 0.64 | 0.06 | 31.84 | 32.62 | 33.95 | 35.44 | 36.95 | 38.34 | 39.17 |
| 17.8 | 0.65 | 0.06 | 31.85 | 32.63 | 33.96 | 35.45 | 36.97 | 38.35 | 39.18 |
| 17.9 | 0.66 | 0.06 | 31.86 | 32.64 | 33.97 | 35.46 | 36.98 | 38.36 | 39.20 |
| 18.0 | 0.68 | 0.06 | 31.87 | 32.66 | 33.99 | 35.48 | 36.99 | 38.37 | 39.21 |
| 18.1 | 0.70 | 0.06 | 31.88 | 32.67 | 34.00 | 35.49 | 37.00 | 38.38 | 39.22 |
| 18.2 | 0.71 | 0.06 | 31.89 | 32.68 | 34.01 | 35.50 | 37.02 | 38.40 | 39.23 |
| 18.3 | 0.73 | 0.06 | 31.90 | 32.69 | 34.02 | 35.52 | 37.03 | 38.41 | 39.24 |
| 18.4 | 0.74 | 0.06 | 31.91 | 32.70 | 34.03 | 35.53 | 37.04 | 38.42 | 39.25 |
| 18.5 | 0.76 | 0.06 | 31.92 | 32.71 | 34.05 | 35.54 | 37.05 | 38.43 | 39.26 |
| 18.6 | 0.77 | 0.06 | 31.93 | 32.72 | 34.06 | 35.55 | 37.06 | 38.44 | 39.26 |
| 18.7 | 0.79 | 0.06 | 31.94 | 32.73 | 34.07 | 35.57 | 37.08 | 38.45 | 39.27 |
| 18.8 | 0.80 | 0.06 | 31.95 | 32.74 | 34.08 | 35.58 | 37.09 | 38.46 | 39.28 |
| 18.9 | 0.82 | 0.06 | 31.96 | 32.75 | 34.10 | 35.59 | 37.10 | 38.47 | 39.29 |
| 19.0 | 0.83 | 0.06 | 31.97 | 32.77 | 34.11 | 35.60 | 37.11 | 38.48 | 39.30 |
| 19.1 | 0.85 | 0.06 | 31.98 | 32.78 | 34.12 | 35.62 | 37.12 | 38.49 | 39.31 |
| 19.2 | 0.86 | 0.06 | 31.99 | 32.79 | 34.13 | 35.63 | 37.14 | 38.50 | 39.32 |
| 19.3 | 0.88 | 0.06 | 32.00 | 32.80 | 34.14 | 35.64 | 37.15 | 38.51 | 39.33 |
| 19.4 | 0.90 | 0.06 | 32.01 | 32.81 | 34.15 | 35.65 | 37.16 | 38.52 | 39.34 |
| 19.5 | 0.91 | 0.06 | 32.02 | 32.82 | 34.17 | 35.67 | 37.17 | 38.54 | 39.35 |
| 19.6 | 0.93 | 0.06 | 32.02 | 32.83 | 34.18 | 35.68 | 37.18 | 38.55 | 39.36 |
| 19.7 | 0.94 | 0.06 | 32.03 | 32.84 | 34.19 | 35.69 | 37.20 | 38.56 | 39.37 |
| 19.8 | 0.96 | 0.06 | 32.04 | 32.85 | 34.20 | 35.70 | 37.21 | 38.57 | 39.38 |
| 19.9 | 0.97 | 0.06 | 32.05 | 32.86 | 34.21 | 35.72 | 37.22 | 38.58 | 39.39 |
